# An interprovincial input–output database distinguishing firm ownership in China from 1997 to 2017

**DOI:** 10.1038/s41597-023-02183-2

**Published:** 2023-05-18

**Authors:** Quanrun Chen, Yuning Gao, Chen Pan, Dingyi Xu, Kun Cai, Dabo Guan, Qi He, Shantong Li, Wanqi Liu, Bo Meng, Zhi Wang, Yang Wang, Xianchun Xu, Peihao Yang, Meichen Zhang, Yuanqi Zhou

**Affiliations:** 1 University of International Business and Economics, Chaoyang District, Beijing, 100029 China; 2 School of Public Policy & Management, Tsinghua University, Haidian District, Beijing, 100084 China; 3 Institute of Quantitative & Technological Economics, Chinese Academy of Social Sciences, Dongcheng District, Beijing, 100732 China; 4 Department of Earth System Science, Tsinghua University, Haidian District, Beijing, 100084 China; 5 Department of Development Strategy and Regional Economy, Development Research Center of the State Council, Dongcheng District, Beijing, 100010 China; 6 Tsinghua China Data Center, Tsinghua University, Haidian District, Beijing, 100084 China; 7 Institute of Developing Economies, Japan External Trade Organization, 3-2-2, Wakaba, Mihama-ku, Chiba-shi, Chiba, 261-8545 Japan; 8 Hubei University of Economics, Wuhan, Hubei Province, 430205 China; 9 George Mason University, Virginia, 22030 USA; 10 School of Economics and Management, Tsinghua University, Haidian District, Beijing, 100084 China; 11 National School of Development, Peking University, Haidian District, Beijing, 100871 China

**Keywords:** Economics, Interdisciplinary studies

## Abstract

Input-Output (IO) data describing supply-demand relationships between buyers and sellers for goods and services within an economy have been used not only in economics but also in scientific, environmental, and interdisciplinary research. However, most conventional IO data are highly aggregated, resulting in challenges for researchers and practitioners who face complex issues in large countries such as China, where firms within the same IO sector may have significant differences in technologies across subnational regions and different ownerships. This paper is the first attempt to compile China’s interprovincial IO (IPIO) tables with separate information for mainland China-, Hong Kong, Macau, Taiwan-, and foreign-owned firms inside each province/industry pair. To do this, we collect relevant Chinese economic census data, firm surveys, product level Custom trade statistics, and firm value-added tax invoices and consistently integrate them into a 42-sector, 31-province IO account covering 5 benchmark years between 1997–2017. This work provides a solid foundation for a diverse range of innovative IO-based research in which firm heterogeneity information about location and ownership matters.

## Background & Summary

Production fragmentation and specialization within China is an important driver of the country’s growth. During this process of production network formation, the Chinese economy also witnessed a significant transformation of trade flow across industries and regions^1^. The multiregional Input-Output (MRIO) model is widely used to assess the impact on growth from a region or sector-specific shock and analyze the structural change in the Chinese economy^2,3^. Recently, province-level and even city-level MRIO tables have been proposed to assist in understanding relevant questions^4–8^. However, in aggregating the production of firms with heterogeneous technologies, the previous subnational MRIO tables were usually compiled under the assumption of homogenous firms within a sector in each region. In addition, most of China’s previously published MRIO tables faced an inconsistency issue caused by the discrepancy between aggregated gross regional production (GRP) and national GDP. In recent years, the Chinese government encouraged the disclosure of official micro data in an effort to promote the development of big data^9^, which made it possible to compile MRIO tables with firm heterogeneity. At the same time, the National Bureau of Statistics of China (NBS) revised China’s historical GRP data based on the 4th national economic census^10^. By incorporating this update, we can mitigate the inconsistency caused by using different sources of regional IO and statistical data to compile interprovincial IO (IPIO) tables.

It is worth emphasizing why it is attractive to compile an IPIO table with heterogeneity across firms and locations to study China’s production network. Unlike small countries, whose production technologies show a low level of variation across different firms and locations, there is strong evidence that an assumption of firm homogeneity may lead to measurement errors and estimation bias^11–13^ because of the significant heterogeneities in production technologies and energy efficiency, technological and financial endowments, and management know-how across firms in China under different types of ownership (e.g., domestically owned and foreign-owned firms) and by geographical location (e.g., coastal or inland areas).

In the definition of Chinese statistics, foreign invested firms can be further categorized into two groups: (i) Hong Kong, Macao, and Taiwan (HMT) invested enterprises; and (ii) other foreign invested enterprises (FIE). Foreign direct investment (FDI) through these firms has played a significant role in China’s rapid industrialization and export miracles. Over the last four decades of China’s opening-up, its FDI inflow experienced steady growth, peaking at 290.9 billion dollars^14^ in 2013. Despite the impact of the global COVID-19 pandemic and geopolitical tension between the US and China, China remained the world’s second-largest recipient of FDI by 2020 (253.1 billion dollars^14^). FIE- and HMT-invested enterprises have contributed to China’s economic growth miracle through various spillover effects, such as branding, sales networks in global markets, technology and managerial know-how transfers, imitation innovations^15^, and human capital accumulation. More importantly, such spillover effects were not distributed evenly across provinces, which further widened the heterogeneities generated by types of firm ownership across China. Distinguishing HMT enterprises from FIEs captures two important features of China’s FDI. First, the regional and sectoral distribution of investment by HMT firms are very different from FDI made by firms from developed countries. Second, the investment objectives of HMT firms are often different than those of FIEs. HMT investment is usually concentrated in export-oriented sectors (vertical FDI), while investment made by FIEs is often focused on China’s vast domestic markets^16^. Therefore, separately tracing the production and trade activities of FIEs and HTM firms throughout the evolution of cross-province supply chains is of great importance for understanding the technological and environmental spillover effects along China’s domestic supply chains and China’s future role in global supply chains.

Several studies measuring domestic value added or carbon emissions in China’s production and trade have explicitly considered heterogeneity across firm types and trade regimes^17–21^. National IO tables based on firm size and ownership types have also been compiled^22–24^. To the best of our knowledge, studies that combine both firm and regional heterogeneities in Chinese economies are rare. The work of Duan *et al*.^25^ was the only to have an MRIO table that captured the firm heterogeneity within a sector in each region in the literature. However, their MRIO tables only distinguished processing and ordinary trade activities and covered 8 regions and 17 sectors. Currently, despite the high demand in the global research community, there is no IPIO table for China that incorporates firm ownership information. This study intends to fill this gap by utilizing the increasingly available micro data.

Using the economic census, industrial firm surveys, product-level customs statistics, and firms’ value added tax (VAT) invoice data, we compiled a new set of IPIO tables for mainland China with separate information on domestically owned, HMT-owned, and foreign-owned firms within each industry in every province. This set of tables combined the strengths of IO tables and national account statistics with firm-level micro data, covering 42 sectors, 31 provinces, and five benchmark years between 1997 and 2017. This new IPIO database has the following special features:

1. All IPIO tables are benchmarked to the up-to-date national account statistics published by the NBS of China.
2. The database consistently identifies firm and regional heterogeneities by dividing each province/industry pair in the calibrated IPIO tables by firm ownership. The types of firm ownership are defined by the share of a set of major economic indicators at the province/industry level, which are estimated from firm-level micro data.
3. The link between micro data and aggregate statistics (e.g., sector-level IO tables and national account statistics) is based on a set of systematically developed concordances among various national and international industrial and product classifications.
4. Firm VAT invoices at the transaction level are used to estimate the interprovincial trade flows.
5. The data production process is transparent. The final datasets are duplicable by readers based on a set of well-documented data files, concordances, and computer codes.

It is worth briefly highlighting how these features can benefit future IO-based research. Feature 1 reduced the inconsistency between the sum of GRP and GDP. Unlike the provincial data reported by local governments in each provincial single IO table (SRIO), GRP data estimated by NBS attempt to correct the bias of local statistics that local officials have more incentives to misreport^26,27^. More importantly, NBS revised China’s historical GDP and GRP data based on the latest economy census to guarantee consistency across provinces over time^10,28^. By benchmarking IPIO tables to the most up-to-date national account statistics in each province consistently compiled by NBS, we also enable meaningful comparisons over time using the new IPIO tables.

Feature 2 not only overcomes the shortcomings of the homogenous firm assumption underpinning official IO statistics but also helps us better understand the indirect economic and environmental effects of firm behaviors through interregional or inter-sectoral linkages. For example, recent studies on international trade have shown that only a small fraction of enterprises, especially large firms, directly participate in international trade. However, small and medium-sized enterprises (SMEs) and non-exporters, most of which are domestically owned, may indirectly engage in international trade by providing intermediate goods and services to exporters, especially large multinationals^29,30^. Therefore, using official firm-level survey data without considering the cross-region, inter-industry domestic production network may underestimate the contribution of SMEs to a country’s foreign trade.

Incorporating additional information from micro data into IPIO tables often reveals inconsistencies between micro and macro statistics. Feature 3 reflects our efforts to build a consistent link between micro data and aggregate statistics at the sector and provincial level to mitigate such inconsistency issues.

Feature 4 overcomes several shortcomings in the previous literature and significantly improves the quality of interprovincial trade flow estimates. Most previous studies compiled MRIO tables based on official provincial IO tables and railway freight transportation statistics released by the Ministry of Transport of China^24,25,31,32^. Due to only limited categories of commodities available in the railway freight records, a gravity model based on strong assumptions was used to estimate the interprovincial trade flows. Given that China’s ever-improving highway network now plays a more important role in interprovincial exchanges than in rail transport, such estimation methods have become increasingly inaccurate.

Feature 5 implies that the database could be updated and easily improved when the NBS of China updates its national account statistics or when better firm-level data become accessible.

It is also worth noting that the IPIO tables we compiled in this paper belong to Inter-Region Input-Output (IRIO) tables rather than MRIO tables in the IO literature. These two types of tables are based on two different models (Chenery-Moses vs Isard^33^), thus are different mathematically. The dimensions of an IRIO table are much higher than a MRIO table with the same region/sector classification. MRIO tables guarantee that interregional production and trade flows exactly meet all regions’ supply and demands but stop short of assigning specific intermediate or final uses for interregional trade flows^34,35^. In contrast, IRIO tables include detailed source/destination and supply/use information which require additional data to separate bilateral trade flows into end-use categories that deliver to sector and final users^31^. Therefore, the data requirements in compiling the two type tables are also different. Compiling MRIO tables needs less detailed interregional trade data but rely on assumptions that trade coefficients by product across different end-users are the same; while compiling IRIO tables needs more detailed cross-region trade statistics to estimate trade coefficients by products across different end-users.

Those new features allow researchers to operationally integrate heterogeneities across geographical locations and firm ownerships into varieties of China-related economic, scientific, environmental, and interdisciplinary studies that were not previously possible, thus enabling them to help policymakers and the public better understand the interregional spillover effects of economic growth and environmental impacts along China’s domestic supply chains. We will provide more details about these features in the rest of the paper.

## Methodology and Data Source

Our approach to constructing the new IPIO tables with three firm ownership types for 1997, 2002, 2007, 2012, and 2017 includes two major steps:

1. China’s provincial MRIO tables were first benchmarked to the most recent national account statistics published by NBS of China and then were rebalanced and transformed to IPIO tables by using trade statistics by end use categories and VAT invoice data;
2. We estimated the shares of gross output, value added (VA), exports, and imports by three types of firm ownership at each of the 31 province/42 industry pairs from various micro statistics, then split each industry in the IPIO table by the three types of firm ownership. In this section, we introduce all the data sources used to construct our database and illustrate the detailed procedures on how the new IPIO tables were constructed.

### Data sources used for constructing IPIO tables by the three types of firm ownership

Table 1 lists all data sources used for compiling our new IPIO tables for 1997, 2002, 2007, 2012, and 2017 and specifies their uses in compiling the IPIO tables. The national account statistics, the MRIO tables from the Development Research Center (DRC) of the State Council, and VAT invoice data were used in step 1, which benchmarked provincial IO tables to the most recent national account data and rebalanced our benchmarked MRIO tables. VAT invoice data and trade statistics by three end use categories were then used to convert MRIO tables into IPIO tables. Detailed economic census data, provincial economic census yearbooks of 31 provinces, the annual industrial survey of industrial firms (ASIF), and China fixed assets investment statistical yearbooks were used to estimate the related shares by firm ownership for splitting IPIO tables in the second major step. In addition to those data sources, trade data from China customs and the relevant concordances for mapping the 8-digit Harmonization System Code (HS) to the Broad Economic Category (BEC) and China’s IO (CIO) industrial classification were used in both steps 1 and 2.

**Fig. 1:**
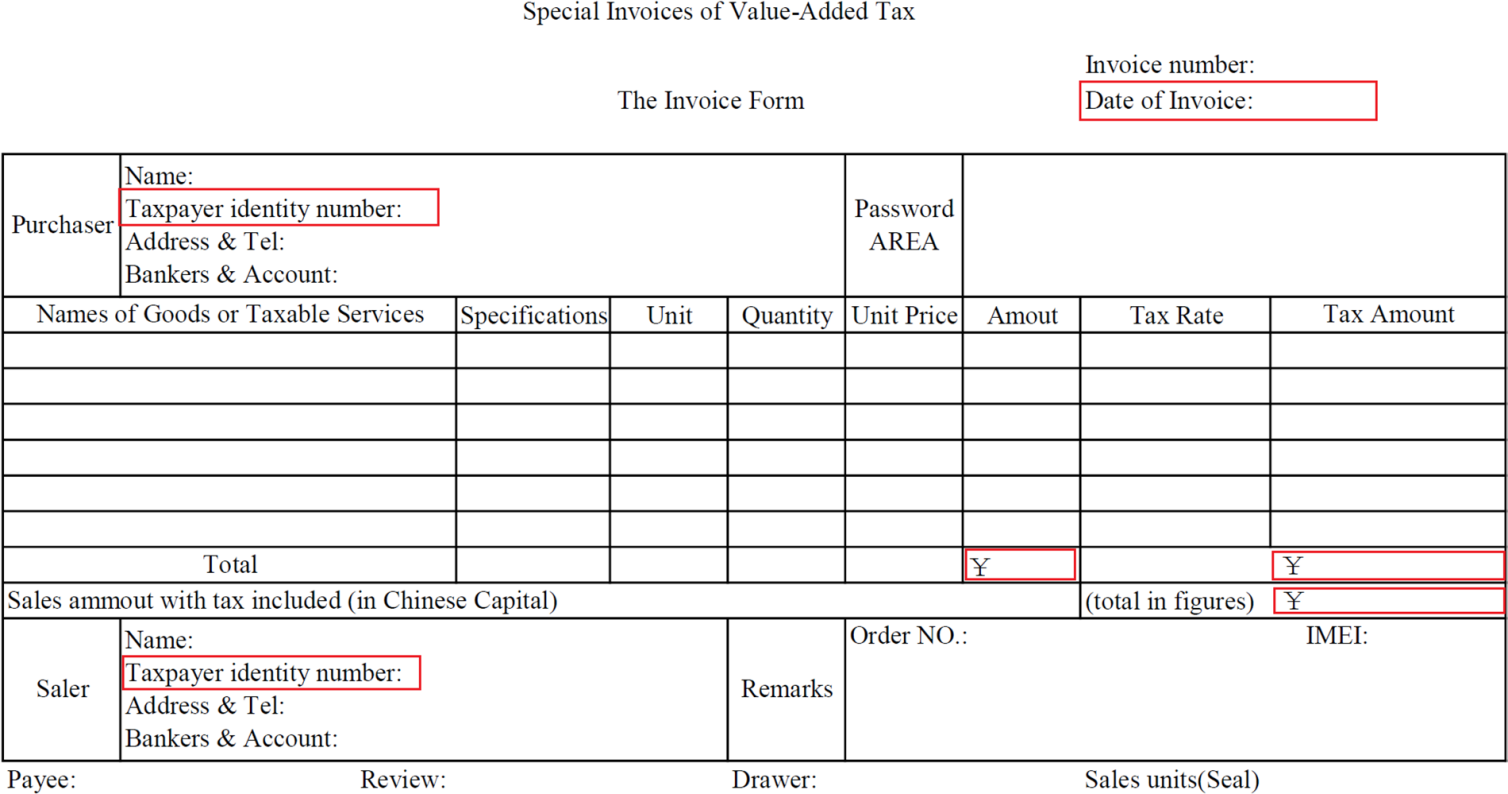
Format of special VAT invoice.
- **China Customs data**
  China Customs data from 1996 to 2017 at the 8-digit HS level were provided by the General Administration of Customs of the People’s Republic of China (GACC). The detailed customs data can be accessed by subscription through guidance on its official website (http://www.customs.gov.cn/customs/302249/zfxxgk/2799825/302274/tjfwzn/2319672/index.html).
- **HS-BEC-CIO concordance**
  The concordance among the HS and BEC (HS-BEC) for 1997, 2002, 2007, 2012, and 2017 was based on the mapping of 6-digit HS codes to BEC developed by the APEC TiVA Technical Group and USITC^43^. The concordance between the 8-digit HS code and China IO sectors for 2007 was published by the NBS of China^44^. Other years (1997, 2002, 2012, 2017) were modified by NBS staff. In addition to those concordances developed by third parties, we also developed several additional concordances in the process of compiling our IPIO tables (more details about concordances can be found in the usage notes).
- **Data for estimating related shares by firm ownership to split the calibrated IPIO tables**

- **Recent national account statistics of China by province**
  This dataset contains GRP data from three accounting approaches (production approach, income approach, and the expenditure approach) covering 31 provinces in mainland China. The production-approach data are classified into 9 sectors: agriculture, forestry, animal husbandry and fishery; industry; construction; wholesale and retail; transportation; warehousing and postal; accommodation and catering; finance; real estate; and others. The income-approach data contain labor compensation, net production tax, depreciation of fixed assets, and operating surplus. The expenditure-approach data are categorized into urban consumption, rural consumption, government consumption, total fixed asset investment, changes in inventories, exports, imports, and interprovincial net outflows.
  China’s national account statistics are updated according to newly available information from time to time. The most up-to-date national account data we used are taken from the online database of NBS of China^36^. Note that even NBS benchmarks the historical GDP and GRP based on the latest census data, the official national account dataset is still not fully internally consistent: the total expenditure-based data at the province level do not exactly equal GRP, and the sum of the GRP does not exactly equal the national GDP.
- **Original MRIO tables and interprovincial trade matrix**
  The original MRIO tables were obtained from the DRC of the State Council. This dataset covers five benchmark years (1997, 2002, 2007, 2012, and 2017)^37–41^ and was constructed using a consistent approach. The data are available in the CDs or via QR code attached with related publications. Specifically, the provincial MRIOs for 1997 and 2017 were products still in process and contributed by two of our coauthors who constructed the DRC MRIO tables. The five MRIO tables are also available in input data files used by our GAMS program to calibrate the IPIO tables. The sector classifications of DRC MRIO tables follow the original classifications adopted in the provincial SRIO tables published by the NBS of China, which slightly differed across the years (40 sectors in 1997, 42 sectors in the other years). The MRIO dataset covers the provinces in mainland China except for Tibet in 1997, 2002, and 2007. DRC estimated bilateral trade flows using partial estimation methods^31^. It consisted of two steps. First, using railway transport data for nine categories of commodities, namely, grain, coal, oil, coke, metal ores, nonmetallic ores, mineral building materials, iron and steel, and fertilizers and pesticides, a gravity model was used to estimate the interprovincial trade flows by sector, and the gravity equations obtained for the nine commodity categories were used to estimate the interprovincial trade flows for other mining and manufacturing sectors that were not directly matched by rail shipments based on the degree of similarity of the commodities.
- **VAT invoices data**
  Our VAT invoice data have three billion invoices per year and cover more than 4 million firms across 31 provinces for 2007, 2012, and 2017. VAT invoice data were obtained from the Golden Tax III system of the State Taxation Administration^42^. Approximately three billion invoices covering four to five million firms were digitized in 2012. The database included detailed transaction information obtained from special VAT invoices (see Fig. 1). These invoices contained detailed information regarding commodity and service transactions, including the taxpayer identification number, company name, location of both the buyer and the seller, type of good or service, quantity, unit price, total amount, and VAT rate.

**Table 1:** Data sources for constructing IPIO tables distinguishing firm ownership.

Data and their sources	Involvement Procedures
National account statistics from NBS	Benchmark provincial IO tables to national account statistics and rebalance
MRIO tables from the DRC	
VAT invoice data	Rebalance the calibrated MRIO tables.
China customs data	Convert calibrated MRIO tables into IPIO tables and split IPIO tables by three types of ownership.
HS-BEC-CIO concordance	
Detailed economic census	Estimate the shares by firm ownership in gross output, value-added and trade for each sector/province pair and split the calibrated IPIO tables by three types of firm ownerships.
Provincial Economic Census Yearbook	
Annual Industrial Survey of industrial firms	
China Fixed Assets Investment Statistical Yearbook	

The references to data sources can be found in the following detailed description.

We combine several data sources to estimate the shares of key variables by firm ownership. Table 2 summarizes the four data sources that were used to identify key economic variables (gross output, value added, exports, intermediate input) by the three firm ownership types. As seen, the fundamental problem in using micro data (e.g., detailed economic census and ASIF) to estimate shares by firm ownership is that none of the data sources could provide all of the required information over the 20-year time period at the province level. Thus, we combined the four data sources to cover all the benchmark years. Based on the four data sources, we computed the shares by firm ownership of gross output, value added, intermediate input, and export delivery for the years 1998, 2004, 2008, 2013, and 2015. We pick the estimated firm ownership shares for the year closest to the benchmark year as the approximation of corresponding shares to split benchmark IPIO tables for 1997, 2002, 2007, 2012, 2017, respectively.

**Table 2:** A summary of data sources for estimating shares by firm ownership.

Data sources	Key coverage	Time and sector coverage
Detailed economic census	Firm level output, value added, intermediate input, export delivery, registered type, registered capital, industry classification code, location code	2004 (only industrial sector available), 2008 (No agricultural sector)
Economic Census Yearbook	Province level output, sales, registered capital	2004, 2008, 2013, 2018 (all sectors)
ASIF	Firm level output, value-added, intermediate input, export delivery, registered type, registered capital, industry classification code, location code, employment	1998–2015 (above scale industrial firms)
China Fixed Assets Investment Statistical Yearbook	Province-sector-level fixed assets investment	1999 (all three main economic sectors, construction, and transportation)

Economic census data (detailed firm level data and summary in yearbooks) and ASIF report the data for the reporting year, while assets investment statistical yearbook reports the data for the previous year.

In 2004, China’s central government conducted the first national economic census covering major Chinese business and industries. The aim was to collect a comprehensive range of accurate economic data to aid economic analysis and policymaking. After 2008, when the second national economic census was conducted, the census was scheduled to be conducted every five years in conjunction with China’s five-year plan. It covers all active firms, irrespective of size or type of ownership. We obtained access to the detailed census data for 2004 and 2008. It encompasses all firms except firms in primary industries in 2008 and all industrial firms in 2004. The number of observations is summarized in Table 3.

**Table 3:** A summary of detailed census data.

Year	Number of observations	Description
2004	1,375,148	All active firms in industrial sectors only
2008	5,228,724	All active firms in all sectors except the primary sector

Industrial sectors cover all sectors in the secondary industry except construction. Detailed census data for 2004 and 2008 were provided by NBS, and they were not free for the public. The national economic census yearbooks for 2004 and 2008^50,51^ provide more details about these two economic censuses conducted by China.

The detailed census data for 2012 and 2018 are still not accessible. Therefore, we used ASIF data to estimate the shares of these major economic variables by firm ownership for industrial sectors in the benchmark years of 1997, 2012, and 2017. A summary is given in Table 4.

**Table 4:** A summary of the annual survey of industrial firms.

Year	Number of observations	Description
1998	165,116	All above-scale firms in industrial sectors only
2013	344,875	All above-scale firms in industrial sectors only
2015	305,498	All above-scale firms in industrial sectors only

Although ASIF covers continuous years from 1998–2015, to keep consistent with the results for other sectors from the census yearbook, we still use the corresponding census year for 2013 to obtain estimations for industrial sectors. ASIF data for 1998, 2013, and 2015 were provided by EPS China Data^52^ (permission required).

The ASIF is also conducted by the NBS and includes similar variables as those in the economic census. There are two key differences between the ASIF data and the detailed census data. First, the ASIF data cover a continuous time period, while the economic census is only conducted every five years. Second, only all state-owned or above-scale industrial firms are included in the ASIF. Above-scale firms are defined by a threshold of sales. Before 2011, the threshold was 5 million yuan, which increased to 20 million yuan after 2011. Even though the ASIF does not include below-scale firms, its detailed information allows us to estimate the shares by types of firm ownership for industrial firms in those years in which detailed census data are not accessible.

Combining detailed census data and ASIF still cannot cover all sectors for all the years needed. Detailed census data cover only a part of industries in China (2004 does not cover the agriculture and service industries, while 2008 does not cover the agricultural sectors). At the same time, ASIF data only cover industrial sectors. To overcome this missing data issue, we used provincial census yearbooks for 2004, 2008, 2013, and 2018 as supplementary data sources for our estimation. After each national economic census, all provincial bureaus of statistics collect the economic data and publish their provincial census yearbook. The format is similar to the national census yearbook but only covers information within each province. Census yearbooks report the output or sales by firm ownership at the industry level. For benchmark year 1997, when census was not conducted, we used China’s Fixed Asset Investment Statistical Yearbook for 1999 as our data source, which included information on national investment in fixed assets in 1998. It provided information on regional (provincial) fixed asset investments by firm ownership in three major industries (primary, secondary, and tertiary). In addition, it also provides information on regional (provincial) fixed asset investments by firm ownership in construction, transport, and real estate. All the provincial census yearbooks and China Fixed Assets Statistical Yearbooks are hard-copy and can be purchased from China Statistics Press^45^.

### Benchmark interprovincial IO tables based on key statistics from China’s national accounts and their rebalancing

**Fig. 2:**
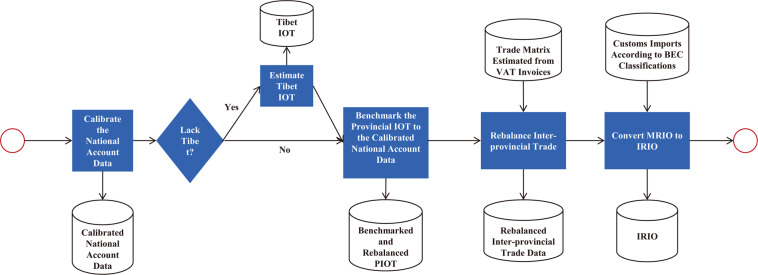
The flowchart of benchmarking and rebalancing the provincial IO tables. The red circles mark the start and end points of the process. Five blue squares represent 5 tasks, linked with the datasets represented by cylinders by arrows showing the flowing direction of data. The blue rhombus indicates whether the Tibet IO table is available. The rest of the arrows show the order of the tasks.

The process of benchmarking and rebalancing the Chinese IPIO tables is summarized by the flowchart (Fig. 2) below. We start by calibrating the national account statistics, followed by benchmarking the provincial IOTs to the calibrated national account data, where the Tibetan IOT was estimated prior to benchmarking if necessary. Then, the interprovincial trade matrices are rebalanced to fit the rebalanced sum of provincial trade in the benchmarked provincial IO tables. Finally, the MRIO tables were converted into IPIO tables. By integrating detailed import statistics by end use and interprovincial transaction aggregated from VAT invoices, we compile China’s IPIO tables that are consistent with the IRIO account in the IO literature.

- **Calibrate China’s national account statistics**
  As mentioned above, the official national account dataset is not internally consistent as a small gap between the sum of GRP and GDP remains for all five benchmark years. Therefore, we need to calibrate the national account statistics before benchmarking the provincial IO tables. We calibrated the GRP by minimizing the squares error with constraints. Equation 1 below shows how the calibration of the production-approach GRP was done. It was also applied to calibrate GRP calculated from the income- or expenditure- approach in a similar way.
  $$\min S=\mathop{\sum }\limits_{r=1}^{31}\mathop{\sum }\limits_{i=1}^{9}{\left({g}_{i}^{r}-g{0}_{i}^{r}\right)}^{2}/\left|g{0}_{i}^{r}\right|$$
  1 $$s.t.\left\{\begin{array}{c}\mathop{\sum }\limits_{i=1}^{k}{\,g}_{i}^{r}=GR{P}^{r}(r=1,\ldots ,31)\\ \mathop{\sum }\limits_{\,r=1}^{31}{g}_{i}^{r}={G}_{i}(i=1,\ldots ,9)\end{array}\right.$$
  where ${g}_{i}^{r}$ represents the value added of region *r*, sector *i*. There are 9 sectors in the production-based value added from NBS. $g{0}_{i}^{r}$ represents the initial value of ${g}_{i}^{r}$. *GRP*^*r*^ is the GRP of region *r*, which is proportionally pre-calibrated to the GDP (see Eq. 2). *G*_*i*_ is the provincial total of sector *i*’s value-added, which is pre-calibrated as Eq. 3:
  2 $$GR{P}^{r}=\left(GRP{0}^{r}/\mathop{\sum }\limits_{r=1}^{31}GRP{0}^{r}\right)\ast GDP$$
  3 $${G}_{i}=\left(G{0}_{i}/\mathop{\sum }\limits_{i=1}^{9}G{0}_{i}\right)\ast GDP$$
- **Estimate the Tibetan IO tables**
  The IO tables for Tibet are not available prior to 2012. Thus, for benchmark years 1997, 2002, and 2007, the tables were constructed based on data from the 2012 IO table for Tibet taken from the MRIO table constructed by the DRC. In terms of sector structure, we used sectoral outputs for Qinghai Province as an approximation for those of Tibet on the grounds that the two provinces share several common geographic and economic characteristics.
  Industrial classification changes across the benchmark years. For 2002, 2007, 2012, and 2017, the IO tables contained 42 sectors, while for 1997, the IO table only included 40 sectors. We concord the IO industry classification backward across the five benchmark years based on China’s Industrial Classification (CSIC) and were able to align sector data between 2012 and previous years. When a sector needed to be split into two or more sectors, the exogenous proportion used was the ratio of sectoral outputs for Qinghai Province in that year.
- **Benchmark the provincial IO tables to the calibrated national account statistics**
  The original MRIO tables were then rebalanced to fit the value-added data at the province level that were calibrated as outlined in step 1. To do so, we used a consistent method across the years. Here, we take the model for 2017 as an example to explain our approach. The model is specified as follows:
  $$S={\rm{\min }}\left(\begin{array}{c}\mathop{\sum }\limits_{i}^{46}\mathop{\sum }\limits_{j}^{51}\mathop{\sum }\limits_{r}^{31}{h}_{ij}^{r}\left({\rm{ln}}{h}_{ij}^{r}-{\rm{ln}}{\bar{h}}_{ij}^{r}\right)\\ +\mathop{\sum }\limits_{ii=1}^{4}\begin{array}{c}\mathop{\sum }\limits_{r}^{31}strin{c}_{ii}^{r}\left({\rm{ln}}strin{c}_{ii}^{r}-{\rm{ln}}strincOb{j}_{ii}^{r}\right)\end{array}\\ +\mathop{\sum }\limits_{jp=1}^{9}\mathop{\sum }\limits_{r}^{31}strpr{d}_{jp}^{r}\left({\rm{ln}}strpr{d}_{jp}^{r}-{\rm{ln}}strprdOb{j}_{jp}^{r}\right)\\ +\mathop{\sum }\limits_{jp=1}^{9}\mathop{\sum }\limits_{r}^{31}strprdMa{x}_{jp}^{r}\left({\rm{ln}}strprdMa{x}_{jp}^{r}-{\rm{ln}}strpr{d}_{jp}^{r}\right)\\ +\mathop{\sum }\limits_{jp=1}^{9}\mathop{\sum }\limits_{r}^{31}strprdMi{n}_{jp}^{r}\left({\rm{ln}}strprdMi{n}_{jp}^{r}-{\rm{ln}}strpr{d}_{jp}^{r}\right)\end{array}\right)$$
  4 $$s.t.\left\{\begin{array}{c}{H}^{r}\cdot {q}_{ctrl}^{r}={x}^{r}\\ \sum _{i}{h}_{ij}^{r}=1;\\ 0\le {h}_{ij}^{r}\le 1;\\ strprd-ad{j}_{max} < {\rm{m}}{\rm{a}}{\rm{x}}\left(vaOb{j}_{prod},vaNB{S}_{prod}\right);\\ strprd+ad{j}_{{\rm{\min }}} > {\rm{m}}{\rm{i}}{\rm{n}}\left(vaOb{j}_{prod},vaNB{S}_{prod}\right);\\ ad{j}_{max}\ge 0;\\ ad{j}_{{\rm{\min }}}\ge 0;\\ inv{t}_{{i}^{\ast }}^{r}=0;\\ e{x}^{r}+pe{x}^{r}\le {x}^{r};\\ pe{x}_{i}-pi{m}_{i}=0;\\ pex\ge 0;\\ pim\ge 0;\\ \mathop{\sum }\limits_{i=43}^{46}\mathop{\sum }\limits_{j=1}^{42}{H}^{r}\cdot {q}_{ctrl}^{r}=GD{P}^{r};\end{array}\right.$$
  The objective function is designed to minimize the distance between the rebalanced data and the original data using the minimizing cross entropy method. The objective function has five terms. The first term is the column structure of the overall table. There are 46 rows (42 sectors and 4 value added items: labor compensation, net production tax, depreciation of fixed assets, and operating surplus) and 51 columns (42 sectors; 5 final use items: urban consumption, rural consumption, government consumption, total fixed asset investment, and changes in inventories; and 4 trade items: exports, imports, interprovincial outflows, and interprovincial inflows). The second term captures the information on GRP calculated from the income approach in four categories. The third term contains the information on GRP calculated from the production approach in nine industries. Both data are from NBS national account statistics and are believed to be more accurate than the GRP calculated from the expenditure approach (this argument is based on the work experience undertaken by one of our coauthors, who oversaw the national account statistics at the NBS for decades.) To keep the sector structure (the related GRP is calculated from the production approach) between the calibrated and the official values aligned as much as possible, we include the fourth and fifth terms in the objective function. The detailed meanings of the notations of the objective function and its constraints are shown in Table 5.

**Table 5:** Notations in the objective function.

Notations	Meaning
*x*^*r*^	${x}^{r}={\bar{x}}^{r}\cdot cV{A}^{r}$, where *x*^*r*^ is the total output of region *r*, and ${\bar{x}}^{r}$ is the initial value of *x*^*r*^. *cVA*^*r*^ is the ratio of the production-approach GRP taken from the calibrated national account data over that of the initial MRIOs ($cV{A}^{r}=vaOb{j}_{prod}^{r}/{\overline{va}}_{prod}^{r}$). Specifically, the total output of Tibet for years 1997, 2002 and 2007 are estimated from the *x*^*Tibet*^ of year 2012, since there are no IO tables of Tibet for these years. For example, ${x}^{Tibet,2007}={x}^{Tibet,2012}\cdot crV{A}^{Tibet,07-12}$,where $crV{A}^{Tibet,07-12}$ is the rate of change of Tibet’s value added between years 2007 and 2012;
${h}_{ij}^{r}$ and ${\bar{h}}_{ij}^{r}$	${h}_{ij}^{r}$ is the column structure of the provincial tables, and ${\bar{h}}_{ij}^{r}$ is the initial value of ${h}_{ij}^{r}$; Specifically, for the “scrap and waste” sector of some provinces, the rate of value added over the total input (VA rate) in the official input-output table of 1997, 2002 and 2007 are equal to 1, which goes against the economic common sense. To deal with this issue, we take the column structure of the province with the highest VA rate as the initial value of the column structure of the few provinces with VA rate of “1” in 2007. For the year 1997 and 2002, since the VA rates of the “scrap and waste” sector in most provinces equal to 1, we take the column structure of the corresponding provinces in 2007 as the initial value.
$inv{t}_{{i}^{\ast }}^{r}$	$inv{t}_{{i}^{* }}^{r}$ is the changes in inventory of sector i*, region *r*. sector i* refer to those sectors with zero changes in inventory in the corresponding national IO tables, which are mostly service sectors;
$strin{c}_{ii}^{r}$ and $strincOb{j}_{ii}^{r}$	$strin{c}_{ii}^{r}=\frac{v{a}_{ii}^{r}}{{\sum }_{ii=1}^{4}\,v{a}_{ii}^{r}}$, where $v{a}_{ii}^{r}$ represents the income-approach GRP term *ii*, region *r*. $strincOb{j}_{ii}^{r}$ is the objective value of $strin{c}_{ii}^{r}$, which is calculated from the calibrated national account data;
$strpr{d}_{jp}^{r}$ and $strprdOb{j}_{jp}^{r}$	$strpr{d}_{jp}^{r}=\frac{v{a}_{jp}^{r}}{\mathop{\sum }\limits_{jp=1}^{9}\,v{a}_{jp}^{r}}$, where $v{a}_{jp}^{r}$ represents the production-approach GRP sector jp (with 9 aggregated sectors), region *r*. $strprdOb{j}_{jp}^{r}$ is the objective value of $strpr{d}_{jp}^{r}$, which is calculated from the calibrated national account data;
$strprdMa{x}_{jp}^{r}$ and $strprdMi{n}_{jp}^{r}$	$strprdMa{x}_{jp}^{r}$ is the adjusted structure of production-approach GRP, whose elements equal to $strprd-ad{j}_{max}$, where $ad{j}_{max}$ is the adjustment value to make $strprd$ less than the larger value between the $strprdOb{j}_{jp}^{r}$ and the official ones (uncalibrated values);Similarly, $strprdMi{n}_{jp}^{r}$ is the adjusted structure of production-approach GRP, whose elements equal to $strprd+ad{j}_{min}$, where $ad{j}_{min}$ is the adjustment value to make *strprd* greater than the smaller value between the $strprdOb{j}_{jp}^{r}$ and the official ones (uncalibrated values).
*pex*_*i*_ and *pim*_*i*_	*pex*_*i*_ and *pim*_*i*_ represent the sum of provincial outflow and inflow by provinces of sector *i*, respectively.

An error item is introduced for the food processing and tabaco industry in 1997 to make the model feasible. The range of the error is ±2% of the sector total output.
  The first constraint is to maintain the row balance of the IO tables. *H*^*r*^ represents the column structure of the IO table for region *r*, whose elements are ${h}_{ij}^{r}$. The term ${q}_{ctrl}^{r}$ is the column sum control, which equals the total output (total input) and the column sum of the calibrated expenditures. Specifically, the column sums of provincial trade inflows and outflows are not controlled, considering that their statistical quality is lower than those of other expenditure items. The 4th to 7th constraints are used for the structure of the production-approach GRP, which is described in Table 5. To avoid re-export, the sum of exports (*ex*^*r*^) and provincial outflows (*pex*^*r*^) should be less than the total output (the 9th constraint). Meanwhile, the regional sum of provincial outflows and inflows (*pim*_*i*_) for each sector should be zero (the 10th constraint) because the sum of outflow from all regions and the sum of inflow from all regions must equal each other for every sector. Finally, the GRP should be equal to the calibrated production side value added from the NBS national account.
- **Rebalance interprovincial trade**

As we mentioned before, most previous research relies on data on rail freight transport to estimate the interprovincial trade flow^24,25,31,32^. However, China’s ever-improving highway network has made road transport less expensive, and road transport now plays a more important role in China’s interprovincial exchanges than rail transport, whose turnover is 6.9 trillion versus 3.3 trillion tons of kilometers in 2021. Thus, the previous interprovincial trade flow estimation method becomes increasingly inaccurate. Therefore, we use unique VAT invoice data at the transaction level from China’s taxation authority as the major data source in this study to estimate China’s interprovincial trade matrix.

The use of VAT invoice data to estimate interprovincial trade linkages has a clear advantage over previous estimation methods based on rail freight data. First, it involves a detailed audit of an enterprise’s VAT invoices and tax payment status via China’s Golden Tax Project, thereby providing accurate digital transaction data. Second, it covers a wide range of goods and services, much broader than what is covered by railway freight data. Third, it is measured in value of the goods and services traded, rather than that in volume as the railway freight data and provides detailed information of the seller and buyer at China’s standard four-digit industry classification (CSIC) for every transaction, thus better satisfying the data needs to compile IRIO tables.

To identify the domestic trade flows between various provinces and sectors, we identified and aggregated firm-level VAT invoices using the following three steps:

1. Select transactions valued at more than five million yuan from the raw VAT invoice records.
2. Extract key information from each VAT invoice. For each VAT invoice, the location at the county and district level, the taxpayer identification number, which included four-digit CSIC, and the total value of the transaction, were collected. Each invoice provided such key information for both purchasers and sellers. The process of adjustment and the structure of the final basic trade flow matrix we developed is shown in Fig. 3.

**Fig. 3:**
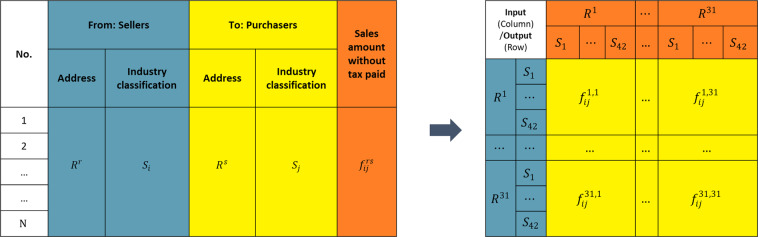
The structure and transformation of Chinese domestic trade flows based on VAT invoices. Suppose that *N* is the total number of VAT invoices in 2012, which can be calculated using the identification number on each special VAT invoice. ${f}_{ij}^{rs}$ refers to the value of transactions from sector *i* in region *r* to sector *j* in region *s*. In this study, for regions *R*^*r*^ and *R*^*s*^ (*r, s* = 1, 2, 3,…, 31), representing China’s 31 provinces, and for sectors *S*_*i*_ and *S*_*j*_ (*i, j* = 1, 2, 3,…, 42), representing the 42 sectors in which economic activities are aggregated from the 4-digit CSIC level.
3. Aggregate the interprovincial trade flow matrix. In theory, the original VAT matrix could be aggregated at the firm level, but in practice, this is hampered by the lack of access to other firm-level data because of commercial privacy concerns. Thus, to enable a comparison of the matrix with existing estimated trade flow matrices, we use the 4-digit CSIC code. The initial aggregated matrix divides economic activity into 58 sectors at the provincial level, and thus, we combined these into 42 sectors based on the classifications used in the IO tables (see Table S1). When the origin and destination shown on the VAT invoice are in the same province, the transaction is considered intraprovincial; otherwise, it is interprovincial.

We integrated the VAT invoice data to re-estimate the interprovincial trade flows. Because the VAT data for service sectors and a few good sectors were sparse, we used the initial interprovincial trade flows in the DRC MRIO tables for these sectors as a supplement.

For the agriculture, mining, manufacturing, and electricity industries, the interprovincial trade matrices estimated from VAT invoice data were used as the initial value to rebalance the interprovincial trade as follows:

$${\rm{\min }}\left(\mathop{\sum }\limits_{s=1}^{31}\mathop{\sum }\limits_{r=1}^{31}{h}_{i}^{sr}\left({\rm{ln}}{h}_{i}^{sr}-{\rm{ln}}{\bar{h}}_{i}^{sr}\right)\right)$$

5 $$s.t.\left\{\begin{array}{c}{H}_{i}pi{m}_{i}=pe{x}_{i}\\ \mathop{\sum }\limits_{s=1}^{31}{h}_{i}^{sr}=1\\ 0\le {h}_{i}^{sr}\le 1\end{array}\right.$$

where ${h}_{i}^{sr}$ refers to the share of the outflow of sector *i*’s product from region *s* to region *r* in the total provincial inflow of region *r*, and ${\bar{h}}_{i}^{sr}$ is the initial value. *H*_*i*_ is composed of ${h}_{i}^{sr}$ multiplied by the sum of provincial inflows (*pim*_*i*_), which should be equal to the sum of provincial inflows (*pex*_*i*_).

For the other sectors, the initial interprovincial trade flows in the DRC MRIO tables were taken as the initial value. The model is the same as that in Eq. 5.

- **Convert MRIO tables to IPIO tables**

Finally, we converted the MRIO tables to IPIO tables. To keep the final IPIO tables consistent with the IRIO account in the IO literature, we integrated the import statistics from China Customs according to the BEC classifications aggregated from the 8-digit HS level prior to the conversion.

First, the import data in the BEC classification were adjusted based on the sectoral imports in the MRIO tables (see Eq. 6):

6 $$i{m}_{i,q}^{r}=im{M}_{i,q}\cdot \frac{im{B}_{i,q}^{r}}{{\sum }_{q=I}^{III}im{B}_{i,q}^{r}}$$

where $i{m}_{i,q}^{r}$ refers to the adjusted imports of sector *i*, category *q*(*q∈*{*intermediates, consumption goods, capital goods*}), and region *r*. Category *q* is defined by BEC (more details about the concordance table between HS, BEC, and China’s IO can be found in the usage notes section). *imM*_*i,q*_ and $im{B}_{i,q}^{r}$ are the sectoral imports in the rebalanced MRIO tables and the imports in the BEC end use categories, respectively. Specifically, if $i{m}_{i,q}^{r}$ is greater than the local demand for the products of sector *i*, category *q*, the excess is proportionally allocated to the other categories.

Second, we calculated the shares of imports, local production, and provincial inflows in the total local use in each province. To calculate the share of imports, we assumed that imports are not used for inventory unless domestic production cannot meet the required changes in inventory. In terms of the allocation of domestic products, a certain share of locally produced products shall be used for local intermediate use, final consumption and capital formation, i.e., Local use except for inventory. We took the share of such local use in total output as the lower bound of the share. Then, the iterative proportional fitting (IPF or RAS) method was used to obtain balanced shares of local production and provincial inflows while the share of imports remained fixed. Here, “balanced” means that the sum of imports, local production, and provincial inflows is equal to total local use based on the constraints of sectoral local production and provincial imports from the rebalanced MRIO tables. The balance is obtained by sector and region. The model used to balance the shares for sector *i*, region *r* is as follows:

$$H=R\cdot \bar{H}\cdot S$$

7 $$s.t.\left\{\begin{array}{c}\mathop{\sum }\limits_{q=I}^{III}hL{p}_{i,q}^{r}\cdot u=L{p}_{i}^{r};\\ \mathop{\sum }\limits_{q=I}^{III}hPi{m}_{i,q}^{sr}\cdot u=Pi{m}_{i}^{sr},(s=1,\cdots \,,31;s\ne r);\\ \left(\mathop{\sum }\limits_{s=1,s\ne r}^{31}hPi{m}_{i,q}^{sr}+hL{p}_{i,q}^{r}\right)\cdot {u}_{q}=\left(1-hI{M}_{i,q}^{r}\right)\cdot {u}_{q},(q=I,II,III);\end{array}\right.$$

where *H* refers to the matrix of shares of imports, local production, and provincial inflows in total local use in sector *i*, region *k* (see Eq. 8). The RAS method was used to determine the appropriate *R* and *S* required to make the initial value of *H* ($\bar{H}$) meet the constraints. The first constraint is to make the sum of local production ($hL{p}_{i,q}^{r}\cdot u$, the change in inventory is deducted, same below) equal to local production in the rebalanced MRIO tables (*Lp*_*i,k*_), where $hL{p}_{i,q}^{r}$ refer to the share of local production, (*i* is the sector index), while *u* is the local end use category index for intermediate use, consumption, and capital formation. Similarly, the second constraint is to make the sum of provincial inflows ($hPi{m}_{i,q}^{sr}\cdot u$) by end use categories equal to the total sectorial provincial inflows in the rebalanced MRIO tables ($Pi{m}_{i}^{sr}$). The third constraint is to make the sum of provincial inflows, local production, and imports equal to the total local use categorized by intermediate use, consumption, and capital formation.

8 $$H=\left[\begin{array}{ccc}hL{p}_{i,I}^{r} & hL{p}_{i,II}^{r} & hL{p}_{i,III}^{r}\\ hPi{m}_{i,I}^{1r} & hPi{m}_{i,II}^{1r} & hPi{m}_{i,III}^{1r}\\ \vdots  & \ddots  & \vdots \\ hPi{m}_{i,I}^{31r} & \ldots  & hPi{m}_{i,III}^{31r}\\ hI{M}_{i,I}^{r} & hI{M}_{i,II}^{r} & hI{M}_{i,III}^{r}\end{array}\right]$$

Then, the rebalanced *H* is used to convert the MRIO tables into IPIO tables assuming that the imports, local production, and provincial inflows used for intermediate use are distributed to the sectors in the same ratios ($hL{p}_{i,q}^{r}$, $hPi{m}_{i,q}^{sr}$, $hI{M}_{i,q}^{r}$). Note that the MRIO table errors in the food processing and tobacco sector in 1997 and those caused by calculation inaccuracy are added to changes in inventory for the goods sectors. For service sectors with no change in inventory in the corresponding national IO tables, the errors (if nonzero) are proportionally allocated to final consumption and fixed capital formation.

### Split IPIO tables by the three types of firm ownership according to their shares of gross output, trade, and value added estimated from firm-level data

After carefully benchmarking the original DRC MRIO tables to the most up-to-date national account statistics and converting them into IPIO tables, we split our IPIO tables by firm ownership estimated from micro data from several sources we described before. Variables for constructing the split IPIO tables include gross output (*x*), exports (*ex*), imports (*im*), value added (*va*), intermediate transaction (*z*), and final use (*f*). All variables at the province-sector level, which are drawn from our calibrated IPIO tables, are further split by using the firm ownership shares estimated from micro data.

- **Gross output, exports, and value added by type of firm ownership**
  The starting point of constructing firm ownership shares is to estimate the shares of gross output, value added, and export delivery by firm ownership. The key information used to distinguish a firm’s ownership type is the firm’s registered type (“*qiye dengji zhuce leixing”* in Chinese) in census or ASIF data. NBS identifies 25 ownership types, including joint ventures between different types of owners. Following NBS’ criteria, we classified these 25 types into three major groups, namely, domestically owned, Hong Kong, Macau, and Taiwan-owned, and foreign-owned. Table 6 shows all 25 detailed ownership types. NBS uses a 3-digit code to classify firms’ ownership types. The firms whose registered ID commenced with “1” are classified as domestic firms. The firms whose registered type ID starts with “2” are classified as “HMT”. The rest of the firms are treated as foreign firms. Firms’ registered IDs are given based on the information on their registered capital. Registered capital can be classified into six types: state, collective, individual, legal person, HMT, and foreign. Following NBS’ classification criteria, the joint venture firm is classified as an HMT or foreign firm if its share of HMT or foreign registered capital is greater than 25%. Otherwise, it is classified as a domestic firm.

**Table 6:** Code of Registration for domestically owned firms.

Registered type ID	Registered type
110	State-owned enterprises
120	Collective enterprises
130	Joint-stock enterprises
141	State-owned associated enterprises
142	Collective associated enterprises
143	State-owned and collective associated enterprises
149	Other associated enterprises
151	Wholly state-owned company
159	Other limited liability company
160	Incorporation
171	Wholly private enterprises
172	Private partnership
173	Private limited liability company
174	Private Incorporation
190	Other domestic enterprises
210	Equity Joint venture enterprises (HMT)
220	Contractual joint venture enterprises (HMT)
230	Wholly HMT investment enterprises
240	HMT Investment limited liability company
290	Other HMT investment enterprises
310	Sino-foreign investment equity joint venture enterprises
320	Sino-foreign investment contractual joint venture enterprises
330	Wholly foreign investment enterprises
340	Foreign investment limited liability company
390	Other foreign investment enterprises
  We proposed a three-step method to estimate the shares of gross output, value added, and export delivery by firm ownership by using detailed census and ASIF data: (1) We used the registered firm type information to identify domestic enterprises. (2) In addition to all the wholly HMT investment enterprises and wholly foreign investment enterprises, we classified joint-venture firms as either HMT- or foreign-owned if at least 25% of their registered capital was HMT- or foreign-owned, respectively. (3) After classifying all firms included in the detailed micro data into one of the three ownership types, we aggregated the outputs, value added, export delivery value, and intermediate inputs at the ownership-province-IO sector level to calculate the shares by firm ownership of the needed economic variables. Because the census data provide information based on China’s standard industry classification (CSIC), CSIC to China’s IO sector (CIO) concordances for each benchmark year were used to aggregate the data at the firm level to China’s IO industries.
  The above 3 steps are the general procedure we used to aggregate the detailed firm-level data to sectors in the calibrated IPIO tables and how the shares of the three firm ownership types were calculated. Ideally, step (1) and step (2) are equivalent since the registered type is consistent with the shares registered capital shares by following NBS’ threshold. However, the registered type and shares of registered capital of some observations are inconsistent. Therefore, we only use registered capital shares for classifying joint-venture firms.
  It is noteworthy that the 3-step method has several issues that require special treatment. First, there were some theoretically inconsistent values reported for key variables, such as negative output, negative employment, and negative registered capital. We assumed that such inconsistencies were the result of measurement errors because there was only a small proportion of inconsistent observations (less than 0.01%). Thus, we simply omitted them.
  Then, value added was not directly reported. Some of the observations record the value of intermediate inputs, and thus, we used the production approach to obtain VA for these observations as follows:
  9 $${\rm{VA}}={\rm{output}}-{\rm{intermediate}}+{\rm{VA}}\;{\rm{tax}}\;{\rm{payable}}$$
  For firms that did not report value added and intermediate inputs, we used the income approach to calculate value added according to Eq. 10:
  10 $${\rm{VA}}={\rm{Depreciation}}+{\rm{Labour}}\;{\rm{compensation}}+{\rm{Net}}\;{\rm{tax}}\;{\rm{of}}\;{\rm{production}}+{\rm{Operating}}\;{\rm{surplus}}$$
  Depreciation is recorded in the detailed census, while labor compensation is calculated by the addition of total wages and benefits plus unemployment insurance. The net production tax is calculated according to Eq. 11:
  11 $$\begin{array}{l}{\rm{Net}}\;{\rm{tax}}\;{\rm{of}}\;{\rm{production}}={\rm{VAT}}+{\rm{Sales}}\;{\rm{tax}}\;{\rm{and}}\;{\rm{extra}}\;{\rm{charges}}+\\ {\rm{Expenses}}\;{\rm{of}}\;{\rm{taxation}}-{\rm{production}}\;{\rm{subsidies}}\end{array}$$
  Here, operating surplus is calculated by the sum of operating profits and production subsidies. Finally, some of the observations did not report information on the firm’s registration type. In the 2008 census, approximately 30% of firms did not report their registration type. For these firms, we skipped the step involving checking their registration type and just identified their type by comparing their HMT- and foreign-owned shares of registered capital with the 25% threshold.
  As mentioned, ASIF data only include above-scale firms, and we need one more step to reduce the bias caused by the exclusion of below-scale firms. We used the type of ownership shares calculated from small above-scale firms, whose features are supposed to be closer to those of below-scale firms, to approximate the shares of below-scale firms. Based on the NBS guidelines, an industrial firm is defined as small or tiny if it employs fewer than 300 people or its annual sales are less than 20 million yuan (only one condition is required to be met, and an above-scale firm can still be a small firm if it meets the requirement). Then, we calculated the weighted average shares by firm ownership, where the weights were the above-scale and below-scale shares computed from the provincial census yearbooks. The calculation of shares by firm ownership $\widehat{{S}_{cen}}$ was as follows:
  12 $$\widehat{{S}_{cen}}={\omega }_{above}{S}_{ind}+{\omega }_{below}{S}_{ind,s}$$
  where *S*_*ind*_ is the share of firm ownership type based on all firms in the ASIF and *S*_*ind,s*_ is the ownership type share based on small industrial firms in the ASIF. *ω*_*above*_ and *ω*_*below*_ are the above-scale and below-scale shares, respectively, of output computed from the provincial census yearbook. This correction cannot solve all selection issues. However, given that below-scale firms constitute only a relatively small portion of the Chinese economy (on average, the contribution to the output from below-scale firms is approximately 10%), the bias of the estimated results after our correction should be acceptable.
  For the missing sectors in detailed census and ASIF data, we used data from provincial census yearbooks to calculate the ownership type shares of gross output in nonindustrial sectors. The following points are noteworthy: First, most provinces do not report sales or output values in the primary and financial industries by registered type but rather by the number of people employed. Considering the large proportion of employment in domestic firms in these two sectors (more than 98% on average), even a large productivity difference between domestic and non-domestic firms does not significantly affect the shares. Therefore, we used these two sectors’ shares of employment by ownership type as a proxy. Second, no production information was reported for government organizations in the census data. Thus, we assumed that all firms in this sector were domestic. Third, for the construction sector, most of the provinces only report output for general contracting firms and specialist contracting firms (i.e., Zongchengbao and Zhuanye Chengbao). These types of firms account for more than 80% of the output of construction firms, and thus, we used their ownership type shares of output as a proxy. Forth, for sectors that only report sales or gross output, all shares were approximated by using the shares of sales or gross output.
  Finally, we used China’s Fixed Asset Investment Statistical Yearbook of 1999 to fill the nonindustrial sectors in the benchmark IO table for 1997. To capture more heterogeneity at the sector level, we also used the Catalogue of Industries for Guiding Foreign Investment (1997 Revision) published by China’s Ministry of Commerce. This catalog shows those industries that are forbidden from accepting foreign investment. For firms in those industries, we assumed that they were domestically owned.
  After estimating the shares of gross output (*sx*), exports (*se*), and value added (*sv*) by firm ownership. We construct the three variables as shown in Table 7:

**Table 7:** A summary of variables estimated from census and industrial firm surveys.

Variables	Relevant formula	Data used for share estimation
$s{x}_{i}^{r}(T)$	${x}_{i}^{r}\left(T\right)=s{x}_{i}^{r}(T){x}_{i}^{r}$	output by different types of ownership
$s{e}_{i}^{r}(T)$	$e{x}_{i}^{r}T=s{e}_{i}^{r}(T)e{x}_{i}^{r}$	export delivery by different types of ownership
$s{v}_{i,k}^{r}(T)$	$v{a}_{i,k}^{r}\left(T\right)=s{v}_{i,k}^{r}(T)v{a}_{i,k}^{r}$	Value-added by different types of ownership

The meanings of superscript and subscript are the same as the previous section. *T*∈{*D, H, F*}°C refers to the ownership type of the corresponding variable. *D* represents domestic, *H* represents Hong Kong, Macao, or Taiwan (HMT), and *F* means foreign. For example, $v{a}_{i,k}^{r}(T)$ is the value added (in subcategory *k*) of industry *i* in province *r*.
- **Imports by types of firm ownership**
  Our approach to constructing imports by different types of ownership requires detailed trade data in addition to census and firm survey data. The products of industry *i* imported from abroad by industry *j* in province *r* by the three types of firms are estimated as follows:
  13 $$i{m}_{ij}^{r}\left(T\right)=s{i}_{j}^{r}\left(T\right)s{m}_{i}^{r}\left(T\right)i{m}_{i}^{r},T\in \left\{D,H,F\right\}$$
  where $i{m}_{i}^{r}$ is total imports of intermediate goods from industry *i* by province *r*. This was calculated from the IPIO tables. $s{m}_{i}^{r}(T)$ is the share of firm type *T* in $i{m}_{i}^{r}$. It is estimated from China’s customs statistics. Therefore, $s{m}_{i}^{r}\left(D\right)i{m}_{i}^{r}$, $s{m}_{i}^{r}\left(H\right)i{m}_{i}^{r}$, and $s{m}_{i}^{r}\left(F\right)i{m}_{i}^{r}$ give the intermediate goods (*i*) imported by firms in province *r* with ownership types *D*, *H*, and *F*, respectively. $s{i}_{j}^{r}\left(T\right)$ is the share of imported intermediate goods (*i*) used by industry *j* of firm type *T*. It was approximated by the intermediate-use share calculated based on China’s economic census and firm survey data. The underlying assumption is that a firm with a high input share also has a high import demand share.
- **Intermediate transactions by type of firm ownership and final use**
  Based on the estimated gross output and exports by different types of firms, the domestic supply by the three types of firms can be calculated. The domestic supply of industry *i* by firm types *D*, *H*, and *F* in province *r* is given by ${x}_{i}^{r}\left(T\right)-e{x}_{i}^{r}\left(T\right)$. The domestic supply share by the three firm types is $s{s}_{i}^{r}\left(T\right)=\frac{{x}_{i}^{r}\left(T\right)-e{x}_{i}^{r}\left(T\right)}{{x}_{i}^{r}-e{x}_{i}^{r}}$. Then, the intermediate products of industry *i* supplied by firms under the three firm types in province *r* consumed by industry *j* in province *s* are estimated by:
  14 $${z}_{ij}^{rs}\left(T\ast \right)=s{s}_{i}^{r}\left(T\right){z}_{ij}^{rs},T\in \left\{D,H,F\right\}$$
  where ${z}_{ij}^{rs}$ is the intermediate products supplied by industry *i* in province *r* consumed by industry *j* in province *s*, obtained from the IPIO tables.
  The final products of industry *i* supplied by three types of firms in province *r* used for final consumption and capital formation in province *s* are estimated by:
  15 $${f}_{i}^{rs}\left(T\right)=s{s}_{i}^{r}\left(T\right){f}_{i}^{rs},T\in \left\{D,H,F\right\}$$
  where ${f}_{i}^{rs}$ is the final products supplied by industry *i* in province *r* used for final consumption and capital formation in province *s*, obtained from the IPIO tables.
  Similarly, we can obtain the intermediate inputs of industry *j* under three firm types in province *s* by ${x}_{j}^{s}\left(T\right)-v{a}_{j}^{s}\left(T\right)$, as well as their shares in total intermediate inputs of industry *j* by $s{i}_{j}^{s}\left(T\right)=\frac{{x}_{j}^{s}\left(T\right)-v{a}_{j}^{s}\left(T\right)}{{x}_{j}^{s}-v{a}_{j}^{s}}$. Then, the intermediate products of industry *i* supplied by firms under the three ownership types in province *s* consumed by industry *j* under the three firm types in province *s* are estimated by:
  16 $${z}_{ij}^{rs}\left(TT\right)=s{i}_{j}^{s}\left(T\right){z}_{ij}^{rs}\left(T\ast \right),T\in \left\{D,H,F\right\}$$
- **Balancing**

Because gross output, value added, and exports by the three firm types are estimated based on firm-level data, we believe that these estimates are reliable and thus keep them unchanged in the balancing procedure. However, the initial estimates of imports, intermediate transactions, and final use by the three firm types are computed based on strong assumptions. This leads to an unbalanced IPIO table at this stage. Next, we update these estimates with constraints of IO account to arrive at a balanced table. To do this, we apply the so-called generalized RAS (GRAS) procedure to the import matrix, intermediate transaction matrix, and final-use matrix with column and row controls (see Table 8).

**Table 8:** Cells to be updated, row controls, and column controls.

(1) Cells in the orange-shaded area are initial estimates of the intermediate transaction matrix, import matrix, and final-use matrix; (2) Cells in the blue- and green-shaded areas are column controls and row controls, respectively; (3) ***i***’ is a row vector with all entries equaling 1; (4) *f*^*r*^ and *f*^s^ are the total final use (final consumption and capital formation) of provinces *r* and *s* for domestic products, computed from the IPIO tables; (5) ***m*** is a column vector of total intermediate imports by industries, computed from the IPIO tables.

Finally, as shown in Table 9, we arrive at the balanced IPIO tables that are split into three types of firms at each province/sector pair. It includes 31 provinces. Three firm types are distinguished for each province (*D, H, F*), and each type of firm engages in production and trade activities in 42 sectors.

**Table 9:** The balanced IPIO tables are split into three types of firms.

			Intermediate Use							Final Demand			Export	Gross output
			Province 1			…	Province 31			Province 1	…	Province 31		
			D	H	F	…	D	H	F	F1 … F5	F1 … F5	F1 … F5		
			1 … 42	1 … 42	1 … 42		1 … 42	1 … 42	1 … 42					
Province 1	D	1	${{\boldsymbol{Z}}}_{1,1}^{DD}$	${{\boldsymbol{Z}}}_{1,1}^{DH}$	${{\boldsymbol{Z}}}_{1,1}^{DF}$	…	${{\boldsymbol{Z}}}_{1,31}^{DD}$	${{\boldsymbol{Z}}}_{1,31}^{DH}$	${{\boldsymbol{Z}}}_{1,31}^{DF}$	${{\boldsymbol{F}}}_{1,1}^{D}$	…	${{\boldsymbol{F}}}_{1,31}^{D}$	${{\boldsymbol{e}}}_{1}^{D}$	${{\boldsymbol{x}}}_{1}^{D}$
		…												
		42												
	H	1	${{\boldsymbol{Z}}}_{1,1}^{HD}$	${{\boldsymbol{Z}}}_{1,1}^{HH}$	${{\boldsymbol{Z}}}_{1,1}^{HF}$	…	${{\boldsymbol{Z}}}_{1,31}^{HD}$	${{\boldsymbol{Z}}}_{1,31}^{HH}$	${{\boldsymbol{Z}}}_{1,31}^{HF}$	${{\boldsymbol{F}}}_{1,1}^{H}$	…	${{\boldsymbol{F}}}_{1,31}^{H}$	${{\boldsymbol{e}}}_{1}^{H}$	${{\boldsymbol{x}}}_{1}^{H}$
		…												
		42												
	F	1	${{\boldsymbol{Z}}}_{1,1}^{FD}$	${{\boldsymbol{Z}}}_{1,1}^{FH}$	${{\boldsymbol{Z}}}_{1,1}^{FF}$	…	${{\boldsymbol{Z}}}_{1,31}^{FD}$	${{\boldsymbol{Z}}}_{1,31}^{FH}$	${{\boldsymbol{Z}}}_{1,31}^{FF}$	${{\boldsymbol{F}}}_{1,1}^{F}$	…	${{\boldsymbol{F}}}_{1,31}^{F}$	${{\boldsymbol{e}}}_{1}^{F}$	${{\boldsymbol{x}}}_{1}^{F}$
		…												
		42												
…	…	..	…	…	…	…	…	…	…	…	…	…	…	…
Province 31	D	1	${{\boldsymbol{Z}}}_{31,1}^{DD}$	${{\boldsymbol{Z}}}_{31,1}^{DH}$	${{\boldsymbol{Z}}}_{31,1}^{DF}$	…	${{\boldsymbol{Z}}}_{31,31}^{DD}$	${{\boldsymbol{Z}}}_{31,31}^{DH}$	${{\boldsymbol{Z}}}_{31,31}^{DF}$	${{\boldsymbol{F}}}_{31,1}^{D}$	…	${{\boldsymbol{F}}}_{31,31}^{D}$	${{\boldsymbol{e}}}_{31}^{D}$	${{\boldsymbol{x}}}_{31}^{D}$
		…												
		42												
	H	1	${{\boldsymbol{Z}}}_{31,1}^{HD}$	${{\boldsymbol{Z}}}_{31,1}^{HH}$	${{\boldsymbol{Z}}}_{31,1}^{HF}$	…	${{\boldsymbol{Z}}}_{31,31}^{HD}$	${{\boldsymbol{Z}}}_{31,31}^{HH}$	${{\boldsymbol{Z}}}_{31,31}^{HF}$	${{\boldsymbol{F}}}_{31,1}^{H}$	…	${{\boldsymbol{F}}}_{31,31}^{H}$	${{\boldsymbol{e}}}_{31}^{H}$	${{\boldsymbol{x}}}_{31}^{H}$
		…												
		42												
	F	1	${{\boldsymbol{Z}}}_{31,1}^{FD}$	${{\boldsymbol{Z}}}_{31,1}^{FH}$	${{\boldsymbol{Z}}}_{31,1}^{FF}$	…	${{\boldsymbol{Z}}}_{31,31}^{FD}$	${{\boldsymbol{Z}}}_{31,31}^{FH}$	${{\boldsymbol{Z}}}_{31,31}^{FF}$	${{\boldsymbol{F}}}_{31,1}^{F}$	…	${{\boldsymbol{F}}}_{31,31}^{F}$	${{\boldsymbol{e}}}_{31}^{F}$	${{\boldsymbol{x}}}_{31}^{F}$
		…												
		42												
Import			${{\boldsymbol{IM}}}_{{\rm{inter}}{\rm{use}}}$							${{\boldsymbol{IM}}}_{{\rm{final}}{\rm{demand}}}$			—	—
Value added	Compensation of employees		${{\boldsymbol{V}}}_{1,1}^{D}$	${{\boldsymbol{V}}}_{1,1}^{H}$	${{\boldsymbol{V}}}_{1,1}^{F}$	…	${{\boldsymbol{V}}}_{1,31}^{D}$	${{\boldsymbol{V}}}_{1,31}^{H}$	${{\boldsymbol{V}}}_{1,31}^{F}$					
	Net taxes on production		${{\boldsymbol{V}}}_{2,1}^{D}$	${{\boldsymbol{V}}}_{2,1}^{H}$	${{\boldsymbol{V}}}_{2,1}^{F}$	…	${{\boldsymbol{V}}}_{2,31}^{D}$	${{\boldsymbol{V}}}_{2,31}^{H}$	${{\boldsymbol{V}}}_{2,31}^{F}$					
	Depreciation on the fixed capital		${{\boldsymbol{V}}}_{3,1}^{D}$	${{\boldsymbol{V}}}_{3,1}^{H}$	${{\boldsymbol{V}}}_{3,1}^{F}$	…	${{\boldsymbol{V}}}_{3,31}^{D}$	${{\boldsymbol{V}}}_{3,31}^{H}$	${{\boldsymbol{V}}}_{3,31}^{F}$					
	Operating surplus		${{\boldsymbol{V}}}_{4,1}^{D}$	${{\boldsymbol{V}}}_{4,1}^{H}$	${{\boldsymbol{V}}}_{4,1}^{F}$	…	${{\boldsymbol{V}}}_{4,31}^{D}$	${{\boldsymbol{V}}}_{4,31}^{H}$	${{\boldsymbol{V}}}_{4,31}^{F}$					
Gross Input			${{\boldsymbol{x}}}_{1}^{D}{\prime}$	${{\boldsymbol{x}}}_{1}^{H}{\prime}$	${{\boldsymbol{x}}}_{1}^{F}{\prime}$	…	${{\boldsymbol{x}}}_{31}^{D}{\prime}$	${{\boldsymbol{x}}}_{31}^{H}{\prime}$	${{\boldsymbol{x}}}_{31}^{F}{\prime}$					

## Data Records

### Balanced IPIO tables split into three types of ownership

IPIO tables split into three types of ownership demonstrate the regional economic structure and interregional supply chains for 31 provinces with 42 sectors (40 sectors for 1997) that split into three types of ownership. They cover China’s economy for five benchmark years: 1997, 2002, 2007, 2012, and 2017. The layout is shown in Table 9. For each year except 1997, the IPIO table contains an intermediate matrix (3,906*3,906) for the 42 sectors in 31 provinces with three firm types. For the year 1997, all dimensions related to the number of sectors are adjusted by 40 instead of 42. For instance, the intermediate matrix is reduced to 3,720*3,720, where 3,720 = 31*40*3. The final demand of each province is similar to other MRIO tables, which consists of 5 categories, including rural household consumption, urban household consumption, government consumption, gross fixed capital formation, and changes in inventories. The final demand matrix contains 3,906*155 elements for each year except 1997. In addition, exports contain 3,906*1 elements measuring the exports for all 42 sectors in 31 provinces by three firm types, while the import matrix contains 42*3,906 elements measuring the imports and their structure from other countries used by all 42 sectors in 31 provinces by three firm types. Value added includes compensation of employees, net taxes on production, depreciation of fixed capital, and operating surplus, with 4*3,906 elements representing four categories of value added for 31 provinces and 42 sectors with three firm types. The above data and related code can be found in the *figshare*^46^.

## Technical Validation

### Comparison of calibrated IPIO tables with major existing datasets

There are several MRIO datasets in China that are publicly accessible. As noted earlier, our IPIO tables started from one of the most widely adopted datasets, the DRC MRIO tables. Therefore, we compared our IPIO tables with three other officially published and widely used Chinese MRIO datasets: the Carbon Emission Accounts and Datasets (CEADs) MRIO Tables (2012 and 2017), the CAS (Chinese Academy of Sciences) MRIO Tables (2012), and the SIC (State Information Center) MRIO Tables (2017). All of these datasets included 42 sectors and 31 provinces. Four variables, the domestic intermediate-use matrix, the sourcing structure of intermediate inputs (shares of imports, interprovincial inflows, and local inputs), the value added rate (at the provincial and sector levels), and the structures of production-approach GRP and income-approach GRP from China’s national account statistics, were involved in the validation.

We followed Steen-Olsen *et al*.^25,47^, Zheng *et al*.^48^, and Canning and Wang^49^ in comparing the four IO variables with the major existing MRIO tables. Three methods were used to compare the IO matrices: the mean absolute percentage error (MAPE), the Isard-Romanoff similarity index (DSIM), and the absolute entropy distance (AED). MAPE and DSIM are “distance” measures, with both measuring the relative distance between two matrices. MAPE values range from 0 to 100, while DSIM values range from 0 to 1. The lower the value is, the greater the similarity between the matrices is. AED is an information-based statistical measure that reflects the difference between the entropies of the two matrices. The closer the AED value is to zero, the greater the similarity between the matrices.

In general, our IPIO tables are similar to the other three MRIO tables in value-added rate and structure of the income-approach GRP, but with two improvements in sourcing structure and sector structures of the production-approach GRP. The comparison shows that the value-added rates in our calibrated IPIOs are very similar to those in the other MRIO datasets not only at the aggregate level (see second row of Table 10) but also at the provincial and sectoral levels (see Tables 11, 12 for details) because we adjusted the total output based on the changes in the NBS’s revised value added at the province/industry level so that the value-added rates, which are more reliable than the total output according to China’s statistics methods, are well kept.

**Table 10:** Comparisons to CEADs MRIO tables, CAS MRIO tables, and SIC MRIO tables.

Variables		2012				2017			
		Dataset	MAPE	DSIM	AED	Dataset	MAPE	DSIM	AED
**VA rate**		**To CEADs**	4.552	0.022	0.000	**To CEADs**	4.087	0.021	0.000
		**To CAS**	3.495	0.017	0.001	**To SIC**	3.288	0.016	0.000
**Sourcing structure of intermediate input**	**Sourcing structure**	**To CEADs**	**21.552**	**0.161**	0.051	**To CEADs**	**27.542**	**0.176**	0.006
		**To CAS**	**24.699**	**0.139**	0.086	**To SIC**	**18.418**	**0.143**	0.013
	**Share of imports**	**To CEADs**	**39.558**	**0.234**	0.013	**To CEADs**	**36.055**	**0.210**	0.040
		**To CAS**	**19.031**	**0.108**	0.019	**To SIC**	**40.525**	**0.219**	0.039
	**Share of inflows**	**To CEADs**	**45.115**	**0.172**	0.036	**To CEADs**	**50.120**	**0.198**	0.018
		**To CAS**	**60.863**	**0.213**	0.037	**To SIC**	**25.386**	**0.123**	0.006
	**Share of local production**	**To CEADs**	13.227	0.078	0.016	**To CEADs**	**17.934**	**0.120**	0.046
		**To CAS**	**16.118**	0.097	0.021	**To SIC**	13.259	0.087	0.030
Intermediate input matrix		**To CEADs**	**43.372**	**0.688**	0.033	**To CEADs**	**59.235**	**0.722**	**0.349**
		**To CAS**	**47.956**	**0.610**	**0.139**	**To SIC**	**51.915**	**0.626**	**0.192**

Numbers in bold indicate lower levels of similarity (MAPE ≥ 15; DSIM ≥ 0.1; AED ≥ 0.1)

**Table 11:** Comparison of value added rates in CEADs MRIO tables, CAS MRIO tables, and SIC MRIO tables for provinces (2012).

Provinces	42 IO sectors						9 National Account sectors					
	To CEADs			To CAS			To CEADs			To CAS		
	MAPE	DSIM	AED	MAPE	DSIM	AED	MAPE	DSIM	AED	MAPE	DSIM	AED
**Beijing**	11.305	0.054	0.035	3.995	0.027	0.002	4.445	0.023	0.002	3.539	0.018	0.010
**Tianjin**	10.544	0.058	0.023	2.709	0.016	0.000	3.571	0.024	0.009	2.616	0.017	0.001
**Hebei**	10.825	0.060	0.018	2.122	0.016	0.000	3.446	0.020	0.010	1.457	0.010	0.004
**Shanxi**	10.260	0.058	0.025	1.439	0.010	0.002	3.496	0.021	0.000	1.407	0.009	0.000
**Inner Mongolia**	9.218	0.053	0.003	2.571	0.016	0.004	2.889	0.016	0.002	2.882	0.019	0.007
**Liaoning**	10.110	0.059	0.027	2.613	0.020	0.002	3.316	0.020	0.009	1.892	0.012	0.003
**Jilin**	11.892	0.067	0.016	3.974	0.022	0.004	5.066	0.032	0.014	3.185	0.020	0.007
**Heilongjiang**	9.494	0.052	0.023	1.733	0.012	0.002	2.857	0.016	0.005	2.151	0.014	0.002
**Shanghai**	10.029	0.055	0.018	3.035	0.018	0.002	3.097	0.020	0.007	2.924	0.018	0.005
**Jiangsu**	9.118	0.051	0.025	2.056	0.014	0.005	3.665	0.019	0.006	2.258	0.013	0.002
**Zhejiang**	9.414	0.055	0.015	2.642	0.018	0.001	3.502	0.021	0.007	1.907	0.011	0.002
**Anhui**	10.479	0.059	0.025	3.646	0.023	0.002	3.605	0.021	0.010	2.399	0.014	0.001
**Fujian**	10.189	0.059	0.018	2.151	0.014	0.004	3.355	0.021	0.010	1.526	0.010	0.003
**Jiangxi**	9.554	0.054	0.019	2.275	0.015	0.002	2.939	0.017	0.003	2.117	0.012	0.000
**Shandong**	10.574	0.065	0.011	2.383	0.020	0.001	3.352	0.019	0.005	1.152	0.007	0.000
**Henan**	10.847	0.064	0.010	3.264	0.022	0.003	3.590	0.022	0.011	2.431	0.016	0.003
**Hubei**	11.988	0.060	0.026	1.968	0.012	0.000	4.121	0.027	0.013	1.937	0.013	0.006
**Hunan**	11.877	0.060	0.023	2.243	0.014	0.003	3.869	0.023	0.010	1.865	0.013	0.005
**Guangdong**	10.626	0.061	0.014	2.191	0.015	0.003	3.988	0.024	0.011	2.007	0.012	0.004
**Guangxi**	11.124	0.058	0.027	1.850	0.013	0.002	2.758	0.015	0.006	1.333	0.009	0.001
**Hainan**	8.896	0.050	0.027	1.553	0.010	0.003	2.628	0.015	0.004	1.815	0.010	0.001
**Chongqing**	10.025	0.059	0.021	2.027	0.013	0.002	3.461	0.022	0.011	1.868	0.013	0.004
**Sichuan**	10.482	0.059	0.034	2.205	0.014	0.004	3.409	0.020	0.009	1.642	0.011	0.004
**Guizhou**	10.108	0.057	0.019	1.545	0.009	0.005	2.877	0.017	0.008	2.082	0.014	0.005
**Yunnan**	8.927	0.052	0.026	1.339	0.007	0.003	3.042	0.016	0.006	1.645	0.009	0.001
**Tibet**	9.708	0.053	0.025	1.910	0.025	0.020	3.022	0.017	0.002	1.262	0.006	0.001
**Shaanxi**	11.410	0.061	0.031	3.285	0.020	0.003	3.091	0.016	0.006	2.041	0.012	0.002
**Gansu**	9.978	0.055	0.011	2.414	0.016	0.001	3.170	0.017	0.002	2.524	0.014	0.000
**Qinghai**	11.435	0.061	0.025	1.778	0.012	0.005	4.513	0.022	0.005	2.783	0.015	0.005
**Ningxia**	10.986	0.056	0.014	2.272	0.018	0.001	2.853	0.016	0.005	1.868	0.012	0.001
**Xinjiang**	10.227	0.060	0.019	2.028	0.013	0.005	3.833	0.023	0.010	1.793	0.013	0.006

Numbers in bold indicate lower levels of similarity (MAPE≥15; DSIM≥0.1; AED≥0.1).

**Table 12:** Comparison of value added rates in CEADs MRIO tables, CAS MRIO tables, and SIC MRIO tables for provinces (2017).

Provinces	42 IO sectors						9 National Account sectors					
	To CEADs			To SIC			To CEADs			To SIC		
	MAPE	DSIM	AED	MAPE	DSIM	AED	MAPE	DSIM	AED	MAPE	DSIM	AED
**Beijing**	11.433	0.059	0.003	3.751	0.021	0.000	5.995	0.031	0.002	2.722	0.014	0.002
**Tianjin**	9.566	0.051	0.010	3.996	0.021	0.002	7.122	0.040	0.003	2.687	0.018	0.008
**Hebei**	9.675	0.050	0.012	2.983	0.016	0.006	7.205	0.037	0.000	2.151	0.009	0.002
**Shanxi**	10.511	0.054	0.008	2.352	0.013	0.002	7.996	0.042	0.002	2.243	0.012	0.002
**Inner Mongolia**	11.005	0.062	0.006	2.676	0.013	0.001	4.842	0.031	0.008	3.110	0.014	0.004
**Liaoning**	10.973	0.055	0.008	2.871	0.017	0.001	6.287	0.032	0.001	1.156	0.007	0.001
**Jilin**	10.723	0.052	0.013	4.958	0.024	0.004	7.532	0.038	0.001	3.317	0.015	0.005
**Heilongjiang**	10.027	0.053	0.000	2.972	0.017	0.003	7.729	0.041	0.002	2.604	0.013	0.001
**Shanghai**	9.932	0.050	0.002	4.609	0.022	0.004	6.460	0.039	0.009	3.398	0.017	0.010
**Jiangsu**	9.713	0.052	0.005	4.180	0.024	0.004	7.391	0.036	0.002	3.115	0.013	0.006
**Zhejiang**	10.838	0.053	0.011	4.569	0.026	0.002	8.065	0.040	0.001	3.917	0.019	0.002
**Anhui**	8.761	0.046	0.003	2.451	0.015	0.002	6.584	0.036	0.003	1.854	0.008	0.001
**Fujian**	9.760	0.053	0.004	3.873	0.020	0.001	8.081	0.046	0.004	5.726	0.033	0.006
**Jiangxi**	9.174	0.047	0.007	2.171	0.014	0.002	7.011	0.036	0.002	1.723	0.010	0.003
**Shandong**	10.483	0.056	0.006	2.611	0.015	0.001	6.803	0.036	0.001	1.691	0.010	0.001
**Henan**	9.851	0.047	0.002	4.067	0.024	0.000	7.550	0.041	0.002	3.521	0.018	0.003
**Hubei**	11.612	0.061	0.007	3.156	0.017	0.002	8.173	0.041	0.003	3.036	0.014	0.000
**Hunan**	10.479	0.052	0.005	1.729	0.010	0.003	6.569	0.032	0.008	1.383	0.008	0.002
**Guangdong**	9.822	0.046	0.007	3.804	0.025	0.005	6.509	0.032	0.006	3.861	0.022	0.004
**Guangxi**	11.131	0.057	0.016	2.095	0.012	0.000	6.558	0.034	0.004	1.474	0.009	0.002
**Hainan**	13.184	0.089	0.018	3.198	0.041	0.020	5.257	0.030	0.000	2.112	0.013	0.004
**Chongqing**	10.463	0.056	0.011	2.098	0.011	0.000	6.361	0.034	0.006	2.042	0.011	0.001
**Sichuan**	10.061	0.053	0.012	1.851	0.011	0.003	6.036	0.030	0.007	1.014	0.006	0.002
**Guizhou**	10.918	0.058	0.000	2.128	0.010	0.001	7.356	0.037	0.000	2.047	0.011	0.001
**Yunnan**	11.124	0.057	0.015	1.680	0.009	0.002	6.359	0.032	0.004	1.047	0.006	0.002
**Tibet**	10.857	0.041	0.004	2.053	0.008	0.001	7.012	0.038	0.002	1.314	0.007	0.000
**Shaanxi**	10.550	0.054	0.001	2.190	0.012	0.000	8.399	0.044	0.005	2.130	0.009	0.003
**Gansu**	11.572	0.061	0.003	4.777	0.024	0.004	8.994	0.046	0.003	6.467	0.031	0.000
**Qinghai**	9.618	0.051	0.002	3.356	0.016	0.000	5.988	0.034	0.004	2.972	0.014	0.004
**Ningxia**	13.646	0.065	0.014	5.489	0.024	0.013	10.443	0.055	0.004	7.226	0.034	0.008
**Xinjiang**	11.342	0.056	0.005	4.963	0.028	0.002	10.919	0.059	0.005	5.312	0.027	0.001

Numbers in bold indicate lower levels of similarity (MAPE ≥ 15; DSIM ≥ 0.1; AED ≥ 0.1).

In addition, since we also tried to make the structure of the income-approach GRP close to that in the NBS national account data, the comparison also shows that our calibrated IPIO data are similar to the other MRIO datasets at the province level (see Table 13 for details).

**Table 13:** Comparison of income-approach GRP structures in CEADs MRIO tables, CAS MRIO tables, and SIC MRIO tables for provinces.

Provinces	2012						2017					
	To CEADs			To CAS			To CEADs			To SIC		
	MAPE	DSIM	AED	MAPE	DSIM	AED	MAPE	DSIM	AED	MAPE	DSIM	AED
**Beijing**	1.179	0.007	0.002	0.673	0.004	0.004	2.744	0.018	0.015	2.154	0.014	0.013
**Tianjin**	2.397	0.016	0.011	2.172	0.015	0.011	4.307	0.026	0.008	3.778	0.021	0.006
**Hebei**	0.393	0.002	0.003	2.791	0.017	0.008	1.000	0.006	0.004	0.961	0.007	0.001
**Shanxi**	3.306	0.019	0.012	3.197	0.020	0.008	0.763	0.004	0.003	2.694	0.016	0.009
**Inner Mongolia**	9.622	0.043	0.016	12.983	0.053	0.028	1.046	0.007	0.004	0.413	0.002	0.003
**Liaoning**	2.433	0.012	0.012	3.539	0.017	0.017	3.009	0.016	0.016	4.903	0.026	0.010
**Jilin**	4.905	0.025	0.016	9.266	0.044	0.029	6.409	0.037	0.007	0.520	0.003	0.001
**Heilongjiang**	2.937	0.018	0.010	4.982	0.021	0.001	4.446	0.024	0.014	5.909	0.032	0.002
**Shanghai**	1.446	0.010	0.006	1.229	0.009	0.004	0.235	0.001	0.000	1.454	0.010	0.008
**Jiangsu**	1.349	0.006	0.002	1.732	0.009	0.001	1.398	0.005	0.003	1.600	0.006	0.002
**Zhejiang**	1.418	0.011	0.009	0.439	0.003	0.001	4.739	0.026	0.026	4.970	0.028	0.027
**Anhui**	0.923	0.005	0.001	3.910	0.020	0.011	4.823	0.025	0.021	5.723	0.031	0.018
**Fujian**	3.098	0.020	0.006	1.389	0.008	0.006	4.100	0.033	0.021	2.178	0.020	0.008
**Jiangxi**	5.849	0.028	0.007	0.529	0.004	0.002	1.285	0.006	0.005	2.615	0.014	0.013
**Shandong**	1.687	0.008	0.001	2.721	0.013	0.001	2.088	0.012	0.010	1.626	0.008	0.001
**Henan**	1.189	0.007	0.003	4.412	0.024	0.009	3.030	0.016	0.013	3.008	0.019	0.004
**Hubei**	2.497	0.011	0.012	1.553	0.011	0.005	0.647	0.004	0.004	2.164	0.010	0.009
**Hunan**	4.944	0.026	0.027	2.201	0.014	0.015	3.215	0.017	0.018	3.229	0.021	0.022
**Guangdong**	2.339	0.016	0.011	1.170	0.009	0.002	2.859	0.018	0.015	0.962	0.007	0.001
**Guangxi**	2.999	0.018	0.015	5.352	0.032	0.022	2.489	0.013	0.012	1.936	0.011	0.006
**Hainan**	1.879	0.012	0.002	1.285	0.007	0.005	3.496	0.022	0.012	0.511	0.003	0.003
**Chongqing**	1.366	0.009	0.003	2.517	0.014	0.009	1.639	0.011	0.006	1.244	0.009	0.003
**Sichuan**	0.551	0.004	0.003	1.795	0.008	0.004	1.295	0.010	0.001	0.595	0.007	0.001
**Guizhou**	5.163	0.033	0.020	2.038	0.019	0.001	2.389	0.020	0.003	1.858	0.013	0.005
**Yunnan**	5.985	0.030	0.033	2.917	0.020	0.016	4.254	0.022	0.026	5.123	0.030	0.026
**Tibet**	2.401	0.019	0.022	1.321	0.010	0.012	1.248	0.010	0.012	1.246	0.011	0.009
**Shaanxi**	0.938	0.008	0.005	1.922	0.011	0.006	1.608	0.009	0.008	0.867	0.005	0.002
**Gansu**	8.228	0.042	0.049	4.271	0.021	0.024	2.426	0.019	0.008	1.878	0.011	0.013
**Qinghai**	12.240	0.063	0.062	16.433	0.081	0.075	3.964	0.032	0.011	2.522	0.014	0.004
**Ningxia**	1.285	0.007	0.005	1.149	0.006	0.004	5.001	0.031	0.028	4.065	0.024	0.027
**Xinjiang**	8.004	0.043	0.058	4.598	0.024	0.030	2.647	0.017	0.023	5.264	0.036	0.048

Numbers in bold indicate lower levels of similarity (MAPE ≥ 15; DSIM ≥ 0.1; AED ≥ 0.1).

One improvement is seen in the sourcing structure of intermediate inputs, which is reflected in the relatively lower level of similarity among the sourcing structure of intermediate input matrices and shares of each source separately (see the middle panel of Table 10). This is because we improved the sourcing structure based on detailed trade statistics aggregated by UN BEC end use categories and use detailed VAT invoice data to estimate interprovincial trade flows, the similarity between our tables and the other tables is expected to be lower. The higher similarity among the other MRIO datasets in the sourcing structure of intermediate input matrices (see second row of Table 14) further reinforced this improvement.

**Table 14:** Comparisons among CEADs MRIO tables, CAS MRIO tables, and SIC MRIO tables.

Variables		CAS vs. CEADs (2012)			SIC vs. CEADs (2017)		
		MAPE	DSIM	AED	MAPE	DSIM	AED
**Sourcing structure of intermediate input**	**Sourcing structure**	10.420	0.115	0.035	13.052	0.102	0.006
	**Share of imports**	**32.768**	**0.225**	0.005	27.442	0.154	0.001
	**Share of inflows**	**16.347**	0.082	0.002	**23.897**	**0.106**	0.024
	**Share of local production**	6.713	0.036	0.006	7.523	0.045	0.016
**Intermediate input matrix**		**34.818**	**0.651**	**0.106**	**48.314**	**0.630**	**0.157**

Numbers in bold indicate lower levels of similarity (MAPE ≥ 15; DSIM ≥ 0.1; AED ≥ 0.1).

Another improvement is reflected in the sector structures of the production-approach GRP. The sector structure of the production-approach GRP in our IPIO tables is almost identical to that in the most up-to-date national account statistics published by NBS of China, reducing the similarities to other MRIO tables at the sector level for both 2012 (see Table 15 for details) and 2017 (see Table 16 for details).

**Table 15:** Comparison of production-approach GRP sector structures in CEADs MRIO tables, CAS MRIO tables, and SIC MRIO tables for provinces (2012).

Provinces	42 IO sectors						9 National Account sectors					
	To CEADs			To CAS			To CEADs			To CAS		
	MAPE	DSIM	AED	MAPE	DSIM	AED	MAPE	DSIM	AED	MAPE	DSIM	AED
**Beijing**	11.693	0.074	0.045	8.904	0.054	0.039	5.207	0.047	0.003	7.162	0.044	0.021
**Tianjin**	**20.998**	0.097	0.050	**24.489**	0.099	0.076	**19.212**	**0.121**	**0.107**	**24.489**	**0.150**	**0.181**
**Hebei**	8.145	0.068	0.028	11.871	0.068	0.053	4.273	0.039	0.000	11.472	0.076	0.081
**Shanxi**	**22.894**	**0.129**	**0.107**	**16.539**	0.071	0.065	**22.866**	**0.145**	**0.237**	**16.338**	**0.140**	**0.157**
**Inner Mongolia**	**24.426**	**0.129**	0.057	**29.884**	**0.163**	0.059	**24.327**	**0.141**	**0.120**	**29.884**	**0.148**	**0.203**
**Liaoning**	12.446	0.075	0.012	12.514	0.053	0.016	7.014	0.047	0.045	10.811	0.074	0.033
**Jilin**	**25.450**	**0.152**	0.041	**35.559**	**0.201**	0.037	**25.252**	**0.153**	**0.179**	**35.559**	**0.195**	**0.253**
**Heilongjiang**	**25.093**	**0.154**	0.040	**21.506**	0.095	0.054	**24.950**	**0.133**	**0.170**	**20.939**	**0.150**	**0.125**
**Shanghai**	**15.633**	0.092	0.078	7.105	0.027	0.021	14.488	0.078	0.066	5.385	0.044	0.018
**Jiangsu**	11.805	0.074	0.004	9.918	0.035	0.010	6.083	0.056	0.005	9.815	0.052	0.097
**Zhejiang**	**16.825**	0.091	0.063	11.345	0.039	0.008	14.296	0.067	0.084	7.925	0.060	0.008
**Anhui**	**17.629**	0.087	0.023	**17.177**	0.067	0.056	13.706	**0.111**	0.043	**16.871**	0.093	**0.133**
**Fujian**	14.540	0.097	0.024	8.350	0.029	0.014	14.345	0.079	0.075	5.932	0.043	0.007
**Jiangxi**	14.430	0.088	0.037	6.582	0.022	0.006	13.581	0.080	0.088	2.952	0.015	0.016
**Shandong**	10.630	0.081	0.016	8.707	0.035	0.016	6.802	0.038	0.038	6.828	0.050	0.044
**Henan**	11.544	0.074	0.016	14.853	0.079	0.016	7.591	0.059	0.025	14.602	0.086	**0.113**
**Hubei**	13.622	0.083	0.022	6.251	0.024	0.002	12.624	0.085	0.039	5.113	0.052	0.039
**Hunan**	14.935	0.093	0.020	10.547	0.040	0.016	14.049	0.078	0.002	9.127	0.066	0.063
**Guangdong**	**17.081**	0.081	0.043	9.856	0.037	0.059	**15.249**	0.081	0.070	8.094	0.053	0.019
**Guangxi**	9.913	0.072	0.043	**17.250**	0.093	0.080	8.568	0.044	0.046	**17.250**	0.077	**0.104**
**Hainan**	7.630	0.076	0.053	5.198	0.025	0.006	5.538	0.046	0.017	5.072	0.032	0.009
**Chongqing**	14.659	0.090	0.066	14.434	0.092	0.027	12.196	0.095	0.005	14.434	0.057	0.083
**Sichuan**	13.217	0.082	0.050	14.408	0.077	0.014	10.056	0.066	0.036	14.408	0.075	0.037
**Guizhou**	12.971	0.080	0.019	13.756	0.040	0.016	11.865	0.061	0.056	11.587	0.094	0.016
**Yunnan**	**15.614**	0.089	0.032	9.494	0.029	0.019	14.967	0.095	0.006	8.453	0.077	0.039
**Tibet**	12.050	0.078	0.097	7.752	0.037	0.057	10.614	0.080	0.044	7.752	0.035	0.007
**Shaanxi**	10.547	0.077	0.028	7.041	0.039	0.006	7.258	0.060	0.030	5.784	0.030	0.046
**Gansu**	**18.541**	**0.107**	0.069	13.024	0.045	0.035	**18.498**	0.087	0.039	11.794	0.097	0.004
**Qinghai**	**25.216**	**0.151**	0.016	**33.689**	**0.202**	0.007	**25.191**	**0.120**	**0.152**	**33.689**	**0.146**	**0.215**
**Ningxia**	**17.020**	0.091	0.029	9.534	0.032	0.017	13.740	0.074	0.051	6.897	0.046	0.001
**Xinjiang**	11.981	0.091	0.043	5.966	0.024	0.023	10.307	0.045	0.043	5.420	0.048	0.006

Numbers in bold indicate lower levels of similarity (MAPE ≥ 15; DSIM ≥ 0.1; AED ≥ 0.1).

**Table 16:** Comparison of production-approach GRP sector structures in CEADs MRIO tables, CAS MRIO tables, and SIC MRIO tables for provinces (2017).

Provinces	42 IO sectors						9 National Account sectors					
	To CEADs			To SIC			To CEADs			To SIC		
	MAPE	DSIM	AED	MAPE	DSIM	AED	MAPE	DSIM	AED	MAPE	DSIM	AED
**Beijing**	14.243	0.087	0.020	8.084	0.044	0.049	7.357	0.072	0.021	6.350	0.051	0.007
**Tianjin**	**26.528**	**0.111**	0.085	**26.473**	0.099	**0.128**	**23.911**	**0.127**	**0.151**	**26.473**	**0.159**	**0.164**
**Hebei**	9.869	0.075	0.000	10.455	0.054	0.050	7.562	0.034	0.038	10.455	0.063	0.060
**Shanxi**	12.698	**0.114**	0.059	12.608	0.054	0.010	8.405	0.073	0.052	12.558	**0.106**	0.068
**Inner Mongolia**	12.169	**0.106**	0.042	2.050	0.012	0.008	9.368	0.079	0.036	1.800	0.019	0.012
**Liaoning**	**17.143**	**0.110**	0.083	14.843	0.057	0.061	14.775	0.079	0.067	13.619	0.089	0.049
**Jilin**	**22.425**	**0.118**	0.041	**27.501**	**0.123**	0.020	**22.299**	**0.107**	0.095	**27.501**	**0.152**	**0.135**
**Heilongjiang**	**26.793**	**0.151**	0.016	**26.132**	**0.118**	0.044	**22.493**	**0.145**	0.091	**26.132**	**0.163**	0.100
**Shanghai**	10.397	0.081	0.034	8.233	0.033	0.013	6.680	0.072	0.050	6.630	0.062	0.010
**Jiangsu**	12.713	0.083	0.014	11.442	0.043	0.039	9.576	0.057	0.026	9.526	0.059	0.037
**Zhejiang**	**20.043**	0.094	0.004	14.751	0.054	0.012	13.871	**0.135**	0.004	13.089	**0.141**	0.041
**Anhui**	**20.107**	0.086	0.042	**19.672**	0.071	0.015	**17.090**	**0.114**	0.063	**19.672**	**0.120**	**0.101**
**Fujian**	**27.730**	**0.116**	0.013	**20.636**	0.068	0.033	**24.252**	**0.213**	0.019	**20.219**	**0.174**	0.076
**Jiangxi**	12.900	0.076	0.019	9.096	0.030	0.017	7.842	0.049	0.011	7.616	0.062	0.022
**Shandong**	10.490	0.078	0.020	9.548	0.044	0.041	6.381	0.045	0.031	9.548	0.072	0.063
**Henan**	**16.192**	0.086	0.009	**16.307**	0.074	0.045	14.674	0.080	0.060	**16.307**	**0.101**	0.084
**Hubei**	12.401	0.091	0.031	10.127	0.034	0.006	6.789	0.059	0.005	8.745	0.073	0.038
**Hunan**	**17.346**	0.090	0.033	**15.487**	0.060	0.059	14.121	**0.100**	0.082	**15.487**	0.092	**0.114**
**Guangdong**	**17.923**	0.094	0.018	8.802	0.036	0.011	14.786	0.085	0.040	5.424	0.037	0.012
**Guangxi**	13.504	0.093	0.023	13.932	0.080	0.059	12.499	0.065	0.032	13.932	0.081	0.044
**Hainan**	10.707	**0.119**	0.066	4.546	0.051	0.024	10.318	0.069	0.002	3.923	0.023	0.010
**Chongqing**	12.494	0.084	0.010	9.382	0.036	0.017	7.826	0.063	0.019	9.382	0.049	0.023
**Sichuan**	12.978	0.085	0.001	8.747	0.027	0.022	7.027	0.056	0.013	8.707	0.070	0.004
**Guizhou**	**18.941**	**0.102**	0.027	**16.099**	0.071	0.008	**18.167**	**0.106**	0.013	**16.099**	**0.116**	0.005
**Yunnan**	**15.617**	0.089	0.049	13.715	0.045	0.042	12.770	**0.105**	0.076	13.651	**0.131**	0.076
**Tibet**	**15.102**	0.074	**0.111**	13.250	0.031	**0.101**	14.355	0.064	0.008	13.241	0.053	0.011
**Shaanxi**	12.079	0.095	0.036	2.156	0.014	0.000	4.442	0.053	0.004	1.010	0.003	0.002
**Gansu**	10.700	0.098	0.016	3.131	0.016	0.004	6.082	0.055	0.007	0.554	0.003	0.003
**Qinghai**	14.599	0.091	0.052	11.045	0.045	0.038	13.371	0.076	0.053	10.561	0.071	0.035
**Ningxia**	**19.823**	**0.109**	0.087	**15.124**	0.047	0.049	**15.911**	**0.104**	0.012	13.708	0.097	0.006
**Xinjiang**	**16.597**	**0.116**	0.079	11.102	0.049	0.052	13.078	0.081	0.021	10.040	0.073	0.002

Numbers in bold indicate lower levels of similarity (MAPE ≥ 15; DSIM ≥ 0.1; AED ≥ 0.1).

There are fewer similarities between the intermediate input matrices than those between the other variables (see last two rows of Table 10). The intermediate input structure between our IPIO tables and the other MRIO tables seems somehow more dissimilar than the dissimilarities among the three MRIO tables compared (see last row of Table 14). This is because the intermediate use matrices reflect the sourcing structure differences among our tables and other tables.

### Consistency checks among share of firm ownership estimates based on economic census and firm survey data on gross outputs, value added, and trade

**Fig. 4:**
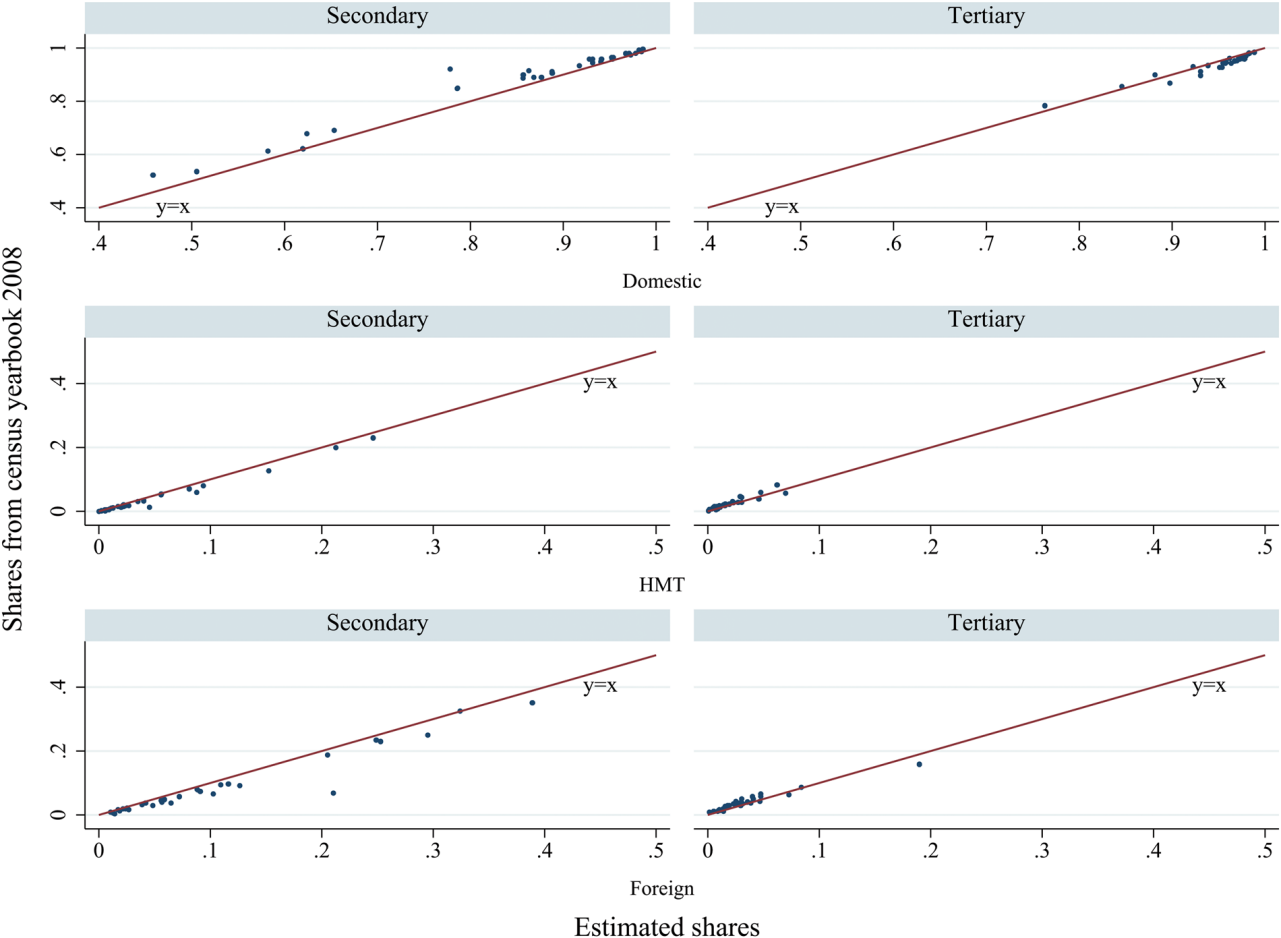
Estimated shares and shares from the 2008 census yearbook. The figure compares each type of estimated shares (domestic, HMT, foreign) with its corresponding share from the census yearbook. The tertiary industry here excludes public management and social security because of the lack of data in census yearbooks.

Given the high degree of accuracy in the provincial/sector-level data drawn directly from the official census yearbook, our consistency checks mainly focused on the consistency between the aggregated micro-level results from our estimates and the aggregated province/sector results reported in the official census yearbook. Figure 4 shows the main results of the comparisons of the detailed 2008 census data by different types of firm ownership for each province. We compared the estimated shares of output for the different firm types in each province with those calculated using the official provincial census yearbook. Following the NBS’s definition of the three major industries, we aggregated the output of the mining industry, manufacturing industry, production and supply of electricity, steam, gas, and water industries, and construction industry by the three firm ownership types to obtain the shares of output by ownership type for the secondary industry. Similarly, we aggregated all service sectors except public organizations to obtain the shares by firm ownership type for the tertiary industry. Overall, the estimates were a good fit with the results from the official census yearbook across the provinces for both industries.

**Fig. 5:**
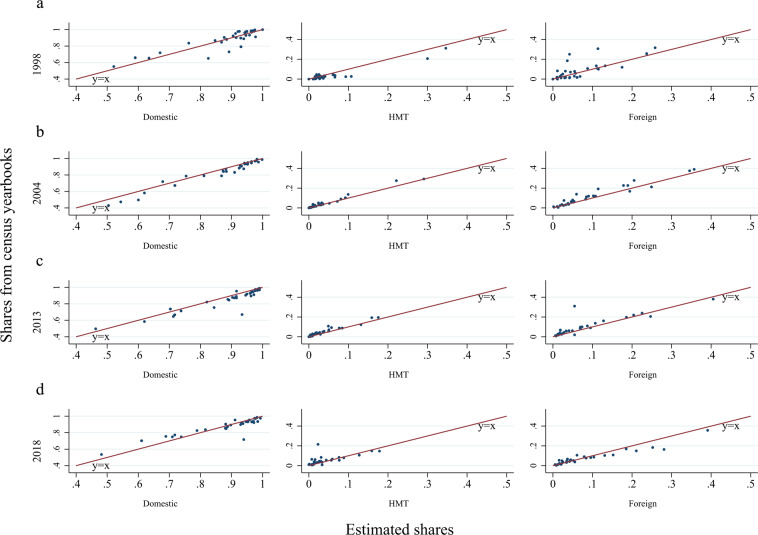
Comparison estimated secondary industry shares for 1998, 2004, 2013 and 2018 with the shares from provincial census yearbooks. All shares are for the secondary industry. For the year 1998, we used the shares from China Fixed Assets Investment Statistical Yearbook instead.

For the rest of the years, consistency at the aggregated level is maintained for the tertiary industry because the shares are directly estimated using aggregated data from the official census yearbooks, so we only checked the aggregated results for the detailed sectors in the secondary industries. Since there were no official census was conducted in 1998, we used the shares of fixed investment by type of ownership as a proxy for comparison. Figure 5 summarizes the consistency check results for the rest of the benchmark years. It shows that there is consistency between the official aggregate shares from yearbooks and our estimates based on micro-level data. The first panel in Fig. 5 shows notable more discrepancy between the estimated results from microdata and the shares from census yearbooks; it is because we used the shares of fixed investment as a proxy. Even so, for most of the provinces, the estimated shares from microdata are still consistent with the shares from the China Fixed Assets Investment Statistical Yearbook.

### Consistency check of the split and rebalanced IPIO tables with firm ownership information and benchmark data

The major regional account data at the province/industry level in the split IPIO tables with three types of firm ownership and in the calibrated IPIO tables are identical because we used the data from the calibrated IPIO tables as strict aggregation constraints to compile the tables with three types of firm ownership. The regional account data include the following:

1. Gross output at the province/industry level
2. Value added at the province/industry level, both for overall value added and four subcategories of value added (employee compensation, net production tax, depreciation of fixed assets, and operating surplus)
3. Total intermediate imports at the province/industry level
4. Imports for final use at the province/industry level
5. Exports at the province/industry level
6. Total final use at the province level (rural household consumption expenditure, urban household consumption expenditure, government consumption expenditure, gross fixed capital formation, and changes in inventories).

The shares for domestically owned firms, firms owned by Hong Kong, Macau, and Taiwan, and firms owned by foreign countries in gross output and export at the province/industry level computed from the split IPIO tables with three types of firm ownership are consistent with the shares by firm ownership estimated from micro data. This is because we use these estimated shares from micro data to split gross output and export by three types of firm ownership and keep them fixed in the balancing procedure. For the estimation of shares in the four subcategories of value added, we encountered missing-data issues. For the 1997, 2002, 2012 and 2017 IPIO tables, we only have micro-level information for industrial firms by three types of ownership and they are consistent with the shares computed from the split IPIO. The information for agricultural firms and service firms are missing. For the 2007 IPIO table, the information for industrial firms and service firms are available, but the information for agricultural firms is missing. To solve this problem, we used the shares of overall value added by the three firm ownership types as a proxy for the shares of value added at the subcategory-level.

## Usage Notes

The five benchmark IPIO tables with three types of firm ownership demonstrate the changes in the production and trade pattern among different sectors and regions over 20 years and can be used to analyze provincial economies within China as a tool for both national and regional economic analysis. Furthermore, by including additional columns such as energy use, carbon emissions, water consumption, air pollution, and employment, these benchmark IPIO tables can be used to undertake extensive China-related research on many economic and environmental issues.

In addition to the IPIO tables, our published datasets include related concordances, relevant input data files, and computer code to generate the IPIO tables. Although these datasets are assembled to generate our IPIO tables with the 3 types of firm ownership, they can also be widely used in research on a variety of China-related issues.

1. Concordances. Three sets of detailed concordance tables were developed to serve as bridges to aggregate the trade data from China Customs and micro data from economic censuses/annual industrial firm surveys to China’s IO industries.
  The first set concordance is among the HS, BEC, and China’s IO industries (HS-BEC-IO), which is based on the mapping of 8-digit HS codes to the CHN IO sectors developed by the NBS of China (see the *NBSHS8toIOsector* files in the concordance folder). This set includes tables for each of the five benchmark year, (1997, 2002, 2007, 2012, and 2017). Based on the mapping between BEC categories and the HS subheadings from the UNSD, which is further modified by industrial specialists at the US International Trade Commission (see *USITC-BEC-HSrev.xls* in the concordance folder) and has been used at the APEC-TiVA project led by both the US and China with the participation of most APEC economies, we were able to aggregate the trade data into three end-use categories: consumption goods, capital goods, and intermediate goods. Imports with China Custom trade codes of 20 (Equipment for processing trade), 25 (Equipment/Materials investment by foreign-invested enterprise), or 35 (Equipment imported into Export Process Zone) were classified as capital goods, and those with codes of 14 (Process & assembling) or 15 (Process with imported materials) were classified as intermediate goods. Concordances of 8-digit HS to China’s IO sector (CIO) for each of the 5 benchmark IO tables are shown as follows Table 17:

**Table 17:** A summary of the concordance of HS to CIO.

Year	HS code	Detailed CIO	CIO
1997	HS1996	124	40
2002	HS2002	122	42
2007	HS2007	135	42
2012	HS2012	139	42
2017	HS2017	149	42
  The second set of concordance tables presents the mapping between the CSIC and IO data and contains five tables, one for each benchmark year. This mapping was undertaken to aggregate the firm-level data in economic censuses and annual surveys of industrial firms (classified by the China System of Standard Industry Classification, CSIC) to China’s IO sector classification for comparison with the International Standard Industrial Classification. For each benchmark year, both the aggregated and detailed IO sectors were mapped to the four-digit CSIC code, as shown in Table 18.

**Table 18:** A summary of the concordance of CSIC to CIO.

Year	CSIC4	Detailed CIO	CIO
1997	GB/T 4754—1994	124	40
2002	GB/T 4754—2002	122	42
2007	GB/T 4754—2002	135	42
2012	GB/T 4754—2011	139	42
2017	GB/T 4754—2017	149	42
  The third set of concordance tables was a chained IO sector concordance among the five benchmark years based on the CSIC to IO sector mapping, containing both detailed and aggregated IO sectors. The groupings of IO sectors, as well as CSIC classifications, have undergone significant changes over the 20-year period. The increasing number of detailed CSIC and IO sectors reflects the refinement efforts of industrial classifications made by the NBS of China, making it very difficult to develop a fully consistent IO sector classification that covers all five benchmark years without losing a significant portion of industrial information in the later benchmark years. Therefore, we developed this backward chained IO sector concordance for database users to aggregate the IO sectors at different benchmark years based on their research needs.
2. Province-IO sector (at both detailed and 42 sector levels) trade data aggregated from China Custom statistics at the 8-digit HS level for 1996–2017, which are distinguished by 5 types of firm ownership (state, whole foreign, joint venture, collective and private) and 3 types of end use categories (intermediates, consumption goods, and capital goods).
3. Share of gross outputs, value-added and export deliveries for domestic-owned, HMT-owned, and foreign-owned firms at each province-sector pair aggregated from detailed census data or census yearbooks.
4. The interprovincial trade matrices aggregated from VAT invoice data for 2007, 2012 and 2017.

## Supplementary information

Table S1. Sectoral correspondence between VAT invoice data and provincial-level IO tables.

## Data Availability

The computer code used to generate the IPIO database with three types of firm ownership for mainland China is based on GAMS and MATLAB. The computer code used to process firm-level micro data and trade statistics is based on STATA. All these codes with detailed instructions have been uploaded in *figshare* provided by Scientific Data^46^. All codes will also be available at https://github.com/abumazan/Interprovincial-IO-database/tree/main after publication. All of the data files used to generate the IPIO tables, except the firm-level data and detailed trade statistics at the product level, are available for public access at *figshare*.
